# Expression of Recombinant Antibodies

**DOI:** 10.3389/fimmu.2013.00217

**Published:** 2013-07-29

**Authors:** André Frenzel, Michael Hust, Thomas Schirrmann

**Affiliations:** ^1^Abteilung Biotechnologie, Institut für Biochemie, Biotechnologie und Bioinformatik, Technische Universität Braunschweig, Braunschweig, Germany

**Keywords:** recombinant antibody, procaryotes, yeast, fungi, insect cells, mammalian cell, transgenic organisms

## Abstract

Recombinant antibodies are highly specific detection probes in research, diagnostics, and have emerged over the last two decades as the fastest growing class of therapeutic proteins. Antibody generation has been dramatically accelerated by *in vitro* selection systems, particularly phage display. An increasing variety of recombinant production systems have been developed, ranging from Gram-negative and positive bacteria, yeasts and filamentous fungi, insect cell lines, mammalian cells to transgenic plants and animals. Currently, almost all therapeutic antibodies are still produced in mammalian cell lines in order to reduce the risk of immunogenicity due to altered, non-human glycosylation patterns. However, recent developments of glycosylation-engineered yeast, insect cell lines, and transgenic plants are promising to obtain antibodies with “human-like” post-translational modifications. Furthermore, smaller antibody fragments including bispecific antibodies without any glycosylation are successfully produced in bacteria and have advanced to clinical testing. The first therapeutic antibody products from a non-mammalian source can be expected in coming next years. In this review, we focus on current antibody production systems including their usability for different applications.

## Introduction

Today, antibodies are used for several applications in research, diagnostics, and therapy. They are used in many standard assays such as immunoblot, flow cytometry, or immunohistochemistry. In addition this, the emerging field of proteome research has a huge need of binders against different protein antigens and splice variants (1, 2). Moreover, recombinant antibodies are used for the diagnosis of different pathogens (3–5) or toxins (6, 7). In the past decade, several antibodies for therapeutic applications have been developed (8, 9), primarily targeting inflammatory or tumor diseases (10). In 2010, sales of approved therapeutic monoclonal antibodies in the USA and EU reached 50 billion US dollars (11).

For the detection of different antigens, polyclonal antibodies are widely used in research and diagnostics. These sera contain a large and diverse amount of different antibodies with unknown specificities. However, polyclonal non-human antibodies may exhibit an immune response in human beings that hampers the therapeutic use for example after snake bites (12). Therefore, the production of monoclonal antibodies (mAbs) by hybridoma technology was a significant milestone (13) for the generation of antibodies for therapeutic use. As this technology is based on the fusion of antibody producing spleen cells from immunized mice or rats with immortal myeloma cell lines, its main obstacle is the inefficient immune response to highly toxic or conserved antigens. In addition, nearly all antibodies which are currently in clinical development are of human-origin or at least humanized in some aspect (9, 14, 15) to prevent immunogenicity. Consequently, transgenic animals, especially mice, have been developed which contain a human immunoglobulin gene repertoire (16, 17) solving the problem of immunogenicity but not the need of an efficient immune response after immunization. Finally, *in vitro* selection technologies such as antibody phage display or ribosomal display provide a solution for the generation of human antibodies (18–22).

**Figure 1:**
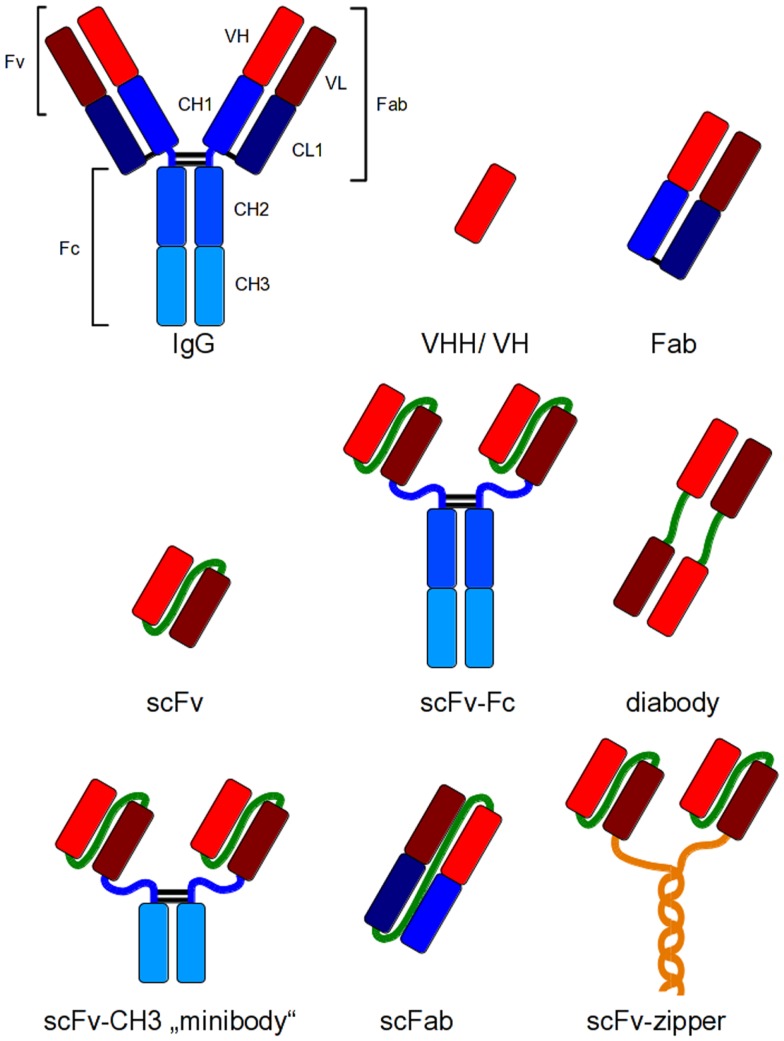
**Recombinant antibody formats for different applications compared to IgG**. Red and dark red: variable regions; blue: constant regions; green: artificial peptide linkers; yellow: dHLX represents amphiphatic helices used for dimerization of scFv fragments.

These new antibody generation technologies have increased the amount of antibodies for different applications and, therefore, also the need of efficient production systems. Immunoglobulin G (IgG) is a heterotetrameric molecule consisting of two heavy and two light chains, respectively, which are connected via disulfide bonds. Heavy and light chains (HC and LC) also contain intramolecular disulfide bonds for stabilization (23). These structural properties require a sophisticated folding apparatus as well as an oxidizing environment for the generation of disulfide bonds. Consequently, many traditionally expression hosts do not provide these mechanisms for efficient production of IgGs. Therefore, smaller antibody fragments have been developed which combine easier production with full antigen binding capacity of an IgG (Figure 1). In addition, the development of smaller fragments was the basis for most of the *in vitro* antibody generation systems (18–22). These antibody fragments can be used for applications, where epitope binding is sufficient for the desired effect including therapeutic applications such as virus neutralization or receptor blocking.

The smallest antigen binding fragment of immunoglobulins maintaining its complete antigen binding site is the Fv fragment, which consists only of variable (V) regions. A soluble and flexible amino acid peptide linker is used to connect the V regions to a scFv (single chain fragment variable) fragment for stabilization of the molecule (24), or the constant (C) domains are added to the V regions to obtain a Fab fragment (Figure 1). Today, scFv and Fab are the most widely used antibody fragments which are produced in prokaryotes. Other antibody formats have been produced in prokaryotic and eukaryotic cells, for example, disulfide-bond stabilized scFv (ds-scFv) (25), single chain Fab fragments (scFab) combining scFv and Fab properties (26) as well as di- and multimeric antibody formats like dia-, tria-, or tetra-bodies (27, 28) or minibodies (miniAbs) comprising different formats consisting of scFvs linked to oligomerization domains like immunoglobulin CH_3_ domain (28), leucin zipper, helix turn helix motif streptavidin, or scFv-scFv tandems (29–31). Bispecific antibody formats combine two different antigen binding domains in one molecule (32–34). The smallest antibody fragments are V_HH_s of cameloide heavy chain antibodies (35) and single domain antibodies (dAb) (36, 37).

For most therapeutic applications, the Fc moiety of an immunoglobulin is essential for the method of action as it mediates the effector functions such as cellular dependent cytotoxicity or the activation of the complement system. Therefore, antibody fragments have been fused to the Fc domain to regain effector functions and avidity (38, 39). Figure 1 depicts some of these antibody formats that have been developed for different applications.

## Antibody Production in Prokaryotic Hosts

### Gram-negative bacteria

*Escherichia coli* is the most important production system for recombinant proteins reaching volumetric yields in the gram per liter scale for extracellular production (40–42). For production of functional antibody fragments, the key to success was the secretion of both V chains into the periplasmic space of *E. coli* where the oxidizing environment allows the correct formation of disulfide bonds and the assembly to a functional Fv fragment (43). This strategy also allowed the first expression of functional Fab fragments in *E. coli* described in 1988 (44).

The production of recombinant antibodies in the reducing cytoplasmic compartment results mostly in non-functional aggregates (45). Recovery of functional antibody fragments from cytoplasmic inclusion bodies by complete denaturation and refolding (46) is often not efficient. Stable cysteine free mutants of some scFvs were successfully produced in the cytoplasm of *E. coli* (47, 48). *E. coli* strains with mutations in the glutathione and thioredoxin reductase in combination with coexpression of cytoplasmic chaperones GroEL/ES, trigger factor, DnaK/J as well as signal sequence-less variants of periplasmic chaperones DsbC and Skp increased the yield of functional Fab (49).

For the production of camelid single domain antibodies (VHH), coexpression of Erv1p sulfhydryl oxidase increased the yield in the cytoplasm (50).

Despite these efforts, most antibody fragments are produced in the periplasm of *E. coli* using N-terminal leader sequences targeting the periplasmic *Sec* pathway (51), for example signal peptides derived from outer membrane protein A (*OmpA*), alkaline phosphatase A *(PhoA*), or pectate lyase B (*PelB*) (52–54). Also the SRP pathway can be used for antibody fragment production (55). After expression, recombinant antibodies are usually isolated from the periplasmic fraction (56, 57) but also from the culture supernatant (58–61).

The yield of functional scFv fragments has been improved by co- or overexpression of GroES/L, peptidyl prolyl-cis,trans-isomerase FkPa, or other folding helper proteins (62–66). Functional expression can also be increased by optimization of cultivation parameters, such as temperature, media, or additives. Here, the optimal parameters are dependent on the individual antibody fragment (58, 67). The production system itself influences the production rate. Very high yields of antibody fragments produced in *E. coli* are mainly provided by high-cell density fermentation in bioreactors: the expression of a hapten-specific scFv produced in a bioreactor (68) lead to yields up to 1.2 g/L compared to 16.5 mg/L yield of the same antibody obtained by optimized shake flask production (69), which can be mostly addressed to the over 100-fold higher cell density in the bioreactor. A recent production system is the LEX bubble column bioreactor. Yields in the LEX system of an anti-MUC1 scFv was ∼30–40× higher and yields of an anti-lysozyme antibody was about 2× higher compared to shake flask incubation (67, 70). *E. coli* strain optimization, e.g., plasmid stability, can additionally improve production yield (71).

The Fab format requires expression, periplasmic transport, correct folding, and assembly of two different polypeptide chains. Among the different vector formats and arrangements, bicistronic vectors with the first cistron encoding the light chain and the second cistron encoding the Fd fragment are optimal (56). Even aglycosylated full-size IgGs were successfully produced in *E. coli* (72, 73). In our view, the raison d’être of complete IgG production in *E. coli* is doubtful.

Cell wall-less L-forms of the Gram-negative bacterium *Proteus mirabilis* were used for the production of miniAbs and scFv (30, 74), but yield of total scFv and of functional scFv were different and ranged from 83 to 127 mg/L of total scFv to just 9–12 mg/L of functional scFv (74). However, quite recently scFv were produced successfully in *Pseudomonas putidas* with a yield of 0.5–3.6 mg/L. Interestingly, production yields were decreased by using scFv genes codon optimized for *P. putidas* (75).

### Gram-positive bacteria

Gram-positive bacteria directly secrete proteins into the medium due to the lack of an outer membrane which could facilitate production of antibody fragments. The Gram-positive bacteria *Bacillus brevis* (76, 77), *Bacillus subtilis* (78, 79), and *Bacillus megaterium* (80–85) have already been successfully used for the production of different antibody fragments. In addition, *B. megaterium* does not produce alkaline proteases and provides high stability of plasmid vectors during growth allowing stable transgene expression during long term cultivation in bioreactors (86).

Lactobacilli are also tested for antibody production and are “generally regarded as safe” (GRAS) microorganisms. To date, two lactobacillus strains were used for the production of scFvs, *Lactobacillus zeae*/*casei* (87, 88), and *Lactobacillus paracasei* (35, 89). The GRAS status of lactobacilli allows their direct use for oral application for example for production of anti-*Streptococcus mutans* antibody fragments to prevent tooth decay (88).

## Eukaryotic Hosts Used for Antibody Production

### Yeasts

Eukaryotic cells have developed an advanced folding, post-translational, and secretion apparatus which enhances the secretory production of antibodies, including full immunoglobulins compared to bacteria. Yeasts combine the properties of eukaryotic cells short generation time and ease of genetic manipulation with the robustness and simple medium requirements of unicellular microbial hosts. Moreover, yeasts have been used for fermentation in food production for several millennia in human history; they do not produce bacterial endotoxins and have gained the GRAS status paving the way toward production of therapeutic proteins (90, 91). *Pichia pastoris* represents the major yeast strain used for recombinant antibody production (92). Other yeasts like *Saccharomyces cerevisiae*, *Hansenula polymorpha*, *Schizosaccharomyces pombe* (93, 94), *Schwanniomyces occidentalis*, *Kluyveromyces lactis*, and *Yarrowia lipolytica* (95) have also been described for protein production but have played only a minor role. *P. pastoris* shows overall optimal capacity for the production and secretion of heterologous proteins than *S. cerevisiae* and does not secrete large amounts of its own protein which simplifies the downstream processing. Moreover, *P. pastoris* prefers respiratory growth resulting in high-cell densities of more than 100 g/L dry weight (96). Probably the most prominent feature of *P. pastoris* is the metabolization of methanol as sole carbon source. The alcohol oxidase 1 (AOX1) promoter is strictly controllable by methanol and commonly used for recombinant protein expression. The secretory production of heterologous proteins including antibodies requires an aminoterminal signal sequence targeting the yeast’s secretory pathway. *S. cerevisiae* mating factor alpha (alpha-factor) pre-pro peptide is the most commonly used secretory signal sequence and is followed by appropriate proteolytic cleavage sites sensitive for the Golgi resident endoprotease KEX2 for efficient release of antibodies during secretion, which is often used in combination with ST13 exoprotease sites (97).

Expression of scFv antibody fragments in *P. pastoris* was first shown by Ridder et al. in 1995 (98). Yields for different scFvs ranged from 70 mg/L (99) to 250 mg/L (100). Up to 8 g/L functional scFv were obtained under optimized conditions in bioreactors with coexpression of BiP (101). Llama VHHs achieved over 100 mg/L yield in *S. cerevisiae* even in shake flask cultivation (102). Production of more complex, yet still single-gene-encoded formats such as dimeric scFv-Fc antibodies in *P. pastoris* achieved production levels of 10–30 mg/L (103). Antibody formats encoded by two genes such as Fab and IgG required the fusion of the two different antibody chains to the aminoterminal secretory signal sequence and their cotransformation. The yield of Fabs produced in yeast ranged from 1 to 50 mg/L by shake flask cultivation and up to 0.5 g/L in bioreactors (96).

Limited data concerning full-sized IgG expression in yeast is available. In an early study, a mouse-human chimeric antibody and its Fab fragment were produced in *S. cerevisiae* with a yield of 50–80 μg/L IgG and 200 μg/L Fab, respectively. The chimeric IgG mediated tumor specific binding and ADCC (antibody dependent cellular cytotoxicity) but no CDC (complement dependent cytotoxicity) (104). Using *P. pastoris* up to 1.4 g/L of a human IgG1 could be expressed in a 40-L bioreactor (105).

Lower transformation rates compared to *E. coli* must be considered for antibody library generation rather than for antibody production. Moreover, the frequency of homologous transformation in yeast is higher compared to higher eukaryotes facilitating the process of making stable expression clones. Specific issues of heterologous protein expression in yeast can be circumvented by optimizing gene sequences, for example by avoiding AT-rich stretches which can cause premature transcriptional termination. The productivity of antibody fragments in yeasts was increased by DNA shuffling (106).

Inefficient secretion of larger heterologous proteins (>30 kDa), proteolysis of secreted proteins during high-cell density fermentation, and inappropriate glycosylation of human glycoproteins are serious issues which required engineering of yeast strains. Overexpression of the chaperone immunoglobulin binding protein (BiP) or protein disulfide isomerase (PDI) in *S. cerevisiae* increased scFv secretion titers twofold to eightfold, with an average yield of 20 mg/L in shake flask culture (107). Yeasts tend to hyperglycosylate heterologous proteins even at positions not glycosylated in the native mammalian host, which can influence activity of antibodies and is a potential source of immunogenicity or adverse reactions in human patients. *P. pastoris* exhibits much lower hyperglycosylation than *S. cerevisiae*, and its *N*-linked carbohydrate structures are already similar to the mammalian high-mannose core unit Man_5–6_GlcNAc_2_ (108). Moreover, genetically modified glyco-engineered *P. pastoris* strains have been generated which produce humanized glycosylation patterns (109–113). The therapeutic IgG antibodies produced in glyco-engineered yeast achieved results that were comparable to its counterpart Trastuzumab that has been produced in mammalian cells (114). Unlike IgGs produced in wildtype yeast, those produced in glyco-engineered yeasts were able to mediate antibody-mediated effector functions. Production processes employing glyco-engineered yeasts are currently optimized for commercial antibody production (115) as well as for high throughput screening (116).

### Filamentous fungi

Filamentous fungi of the genera *Trichoderma* and *Aspergillus* have the capacity to secrete large amounts of proteins and metabolites into the medium (117). They are widely used in the food and biotechnological industry, for example *A. niger* for citric acid production. Moreover, *A. niger* (subgenus *A. awamori*) and *Aspergillus oryzae* gained obtained GRAS status. Two promoters are typically used for the expression of antibodies in fungi: the glucoamylase promoter (glaA) (118) and the endoxylanase A promoter (exlA) (119). Antibody chains are usually fused to the aminoterminus of glucoamylase in *Aspergillus* and cellobiohydrolase I in *Trichoderma spec*., respectively, in order to obtain optimal secretion (120). Moreover, protease cleavage sites like KexB are introduced to release the antibody from glucoamylase before secretion (118). Yields of up to 1.2 g/L IgG were achieved in *A. niger* when both antibody chains were fused to glycoamylase. In *Trichoderma reesei*, 150 mg/L of a Fab fragment was obtained when both chains were fused with cellobiohydrolase I increasing yields 100-fold higher than with its natural signal peptide (121). *A. awamori* was used for the production of several scFvs, llama VHHs and antibody enzyme fusion proteins (117, 119, 122). A yield of 73.8 mg/L of an anti-EGFR-VHH was achieved in *A. oryzae* by using a Taka-amylase A signal sequence and 28 amino acids from the aminoterminal region of *Rhizopus oryzae* lipase (123).

Fungal proteases can result in protein degradation which was addressed by deletion mutants. *Chrysosporium lucknowense C1* contains a triple protease deletion (Delta-alp1, Delta-pep4, Delta-alp2) and was successfully used in small-scale productions for screening as well as in high scale bioreactor productions (124).

### Protozoa

Recently, the eukaryotic parasite *Leishmania tarentolae* has been explored as an expression system for different recombinant proteins (125, 126). One major advantage of this expression system is the mammalian-like glycosylation pattern: this protozoa is able to perform O-glycosylation as well as N-glycosylation, which is highly conserved in mammalians (127). Consequently, *L. tarentolae* has been begun to be used for the production of recombinant antibodies: analysis of different signal peptides lead to a protein yield of 2–6 mg/L purified scFv (128).

### Insect cells

Insect cells represent a very versatile eukaryotic expression system. They can be efficiently transfected with insect-specific viruses from the family of *Baculoviridae*, particularly the *Autographa californica* nuclear polyhedrosis virus (AcNPV). Baculoviruses are highly species-specific and are considered as safe for humans, mammalians and plants. Infection of human hepatocytes and mammalian cell lines including stable transduction has been demonstrated in cell culture without evidence of viral replication or gene expression under the control of baculoviral promoters (129, 130). Non-essential baculovirus genes involved in the viral life cycle, like *Polyhedrin*, *P10*, or *Basic* can be replaced by heterologous genes. The flexible viral envelop allows packaging of large heterologous gene sequences of more than 20 kb. Heterologous genes under the control of the strong polyhedron promoter are expressed at levels ranging from 0.1 to 50% of the total insect cell protein. Baculoviral protein expression is normally performed in insect cell lines like Sf-9 and Sf-21 of *Spodoptera frugiperda*, DS2 cells of *Drosophila melanogaster*, or High Five cells (BTI-TN-5B1-4) of *Trichopulsia ni*. High Five cells have certain advantages over Sf-9 cells for recombinant protein expression because they secrete up to 25-fold higher protein levels (131), have a more rapid doubling time, allow quick adaptation to serum-free medium and grow in suspension culture. In contrast, Sf-9 and Sf-21 cells are recommended for producing high-titer viral stocks due to higher transfection efficiency. Recombinant protein production can be performed in small-scale using plates or shake flasks as well as in large scale using Spinner flasks or bioreactors. Important parameters for optimizing baculoviral protein production are multiplicity of infection (m.o.i.), production length (usually up to 96 h), addition of protease inhibitors due to the release of viral proteases, temperature (usually 25–30°C), and media pH (pH 6.0–6.4).

Secreted monomeric anti-phOx scFv were obtained at levels of up to 32 mg/L in a 6-L bioreactor with 10^9^ cells per liter after 72 h with an m.o.i. of 1 (132). Production yields of 6–18 mg/L have been achieved for various IgGs (133). Immunoglobulins produced in High Five cells showed mammalian-like terminal galactosyl residues β(1,4)-linked to the biantennary GlcNAc residues. In contrast, the absence of sialylation, the formation of paucimannosidic structures and the presence of potentially allergenic α(1,3)-fucose linkages are different to mammalian glycosylation (134). Nevertheless, IgGs produced in insect cells were able to mediate effector functions like complement binding (135, 136) and ADCC (137). Insect cell protein expression was improved using protease deficient baculovirus strains or cell lines with additional glycosyltransferase gene modifications to obtain glycosylation patterns comparable to mammalian cell lines (138–141).

Expression of IgGs in insect cells under control of the strong *Polyhedron* promoter resulted in an extensive aggregation, probably caused by overloading the cellular folding and post-translational processing apparatus (142).

Overexpression of the ER resident chaperone binding protein (BiP) significantly enhanced levels of soluble and secreted IgGs in *T. ni* cells (143). Enhanced secretion of IgGs was also achieved by coexpression of protein disulfide isomerase (PDI) or the human cytosolic chaperone hsp70 in *T. ni* cells (138).

Due to strong usage of the cellular metabolism during baculoviral protein expression a high diversity in the post-translational modification was observed. Alternatively to baculoviral expression, insect cells can also be transfected with expression plasmids in a transient or stable manner. Here, usually Schneider 2 (S2) cells of *D. melanogaster* are used. Secretory production requires a signal sequence like the honeybee melittin leader. Stable transfection of *Drosophila* cell lines with monomeric and dimeric antibody fragments resulted in yields of up to 25 μg/mL (144).

Immunoglobulin G production using the baculovirus expression system demonstrated IgG effector function such as complement binding (135, 136). 10 μg/mL of anti-Rhesus D antibody produced in Sf-9 cells mediated lysis of Rh+ red blood cells by ADCC (137).

### Mammalian cells

Today, 60–70% of all recombinant protein pharmaceuticals and 95% of the currently approved therapeutic antibodies are still produced in mammalian cell lines despite relatively high production costs and difficult in handling. However, the advanced mammalian folding, secretion and post-translational apparatus is capable of producing antibodies indistinguishable from those in the human body with least concerns for immunogenic modifications. Moreover, it is also highly efficient for secretion of large and complex IgGs and in combination with the folding and post-translational control it results in high product quality which reduces efforts and costs in the subsequent and more expensive downstream processing steps. The risks of contamination by pathogens or bovine spongiform encephalopathy (TSE/BSE) agents have been eliminated by well-documented Good Manufacturing Practice (GMP) compliant designer cell substrates and chemical defined media without the need of supplementing animal serum components (145). In 2004, mammalian cell culture technology reached production levels of approximately 5 g/L IgGs in Chinese hamster ovary (CHO) cells (146). Today, industrial IgG production levels often exceed 12 g/L as the result of a steadily ongoing progress in mammalian cell culture technology, which is mainly due to improved high producer cell lines, optimized production media, and prolonged production processes at high-cell densities. The highest reported IgG production titer we found was obtained in the human embryonic retinal cell line Per.C6 [Crucell, Leiden, Netherlands, (147)] with 27 g/L. Generally, the productivity of recombinant mammalian cell lines increased from initially 10 pg antibody per cell per day (pcd) in 1986 to about 90 pcd in 2004. Today, the antibody production levels only rarely exceed 100 pcd because higher cellular productivity usually corresponds to lower maximum cell densities in the production process. Producer cell lines have also been genetically engineered regarding product homogeneity, improved metabolism, reduced apoptosis, and inducible cell cycle arrest (148, 149) which allows prolonged production times for almost 3 weeks at high-cell viability and cell densities.

Chinese hamster ovary (CHO) cells are the most common cells applied in the commercial production of biopharmaceuticals. This cell line isolated in the 1950s gave rise to a range of genetically different progeny, such as K1-, DukX B11-, DG44-cell lines and others which differ in protein product quality and achievable yield. In addition, Per.C6 cells, mouse myeloma NS0 cells, baby hamster kidney (BHK) cells and the human embryonic kidney cell line HEK293 received regulatory approval for recombinant protein production. Although glycosylation patterns of mammalian glycoproteins are very similar to that in humans (150), even small differences can influence pharmacokinetics and effector functions of antibodies. Alternative designer cell lines with improved glycosylation patterns have been generated, for example human neuronal precursor cell line AGE1.HN (Probiogen, Berlin, Germany) supporting specific and complex glycostructures for the production of antibodies which require specific post-translational modifications or suffer from instability or susceptibility for proteolysis (151). CHO cell variant Lec13 (Glycotope) also produces human IgG1 with *N*-Linked glycans lacking fucose which improves on Fc-gammaRIII binding and ADCC (152).

#### Stable production of antibodies in mammalian cells

The generation of stable master cell lines is a prerequisite for GMP compliant IgG production in the therapeutic sector in order to guarantee long term production stability. Here, the antibody gene expression cassettes have to be stably integrated into the host cell genome.

Strong promoters like the immediate early cytomegalovirus (CMV) or the cellular elongation factor (EF) 1-alpha promoter and polyadenylation sites from the simian virus (SV) 40 or the bovine growth hormone (BGH) for improved mRNA stability and translation efficiency are usually implemented into the expression vector. Furthermore, splicing of mRNA is known to promote mRNA packaging and transfer into the cytosol in order to stabilize and enhance gene expression as well as to reduce silencing of heterologous transgenes (153, 154). For IgG expression, two different genes must be stably transfected into one cell clone, either by cotransfection or by using bicistronic expression vectors. Bicistronic vectors employing internal ribosomal entry sites (IRES) allow the translation of two or more cistrons from the same transcript (155). The encephalomyelitis virus (ECMV) IRES has shown the highest efficiency in various mammalian cell lines. Mutated IRES derivatives allow the control of translation efficiency in relation to the cap-dependent cistron. The ratio between light and heavy chain has great impact on the secretion level of functional IgGs (156). The long term stability of ECMV IRES containing bicistronic constructs has been demonstrated even in the absence of selection pressure over months (157).

There are different methods to enhance antibody expression by increasing the number of antibody gene copies in the genome through gene amplification. The two major systems on the market are based on dihydrofolate reductase (DHFR) or glutamyl synthetase (GS) selection. Yield and functionality of an IgG1 produced in *dhfr*^−^ CHO and GS-NSO are equivalent (158) and reached 1.8 g/L in GS-NS0 cells (159). However, gene amplification also causes genetic instability, and after removing the selection pressure the yield of antibodies can be reduced again. Moreover, high producer cell lines often contain only a few copies of the antibody genes. For example, up to 2.7 g/L final antibody concentration were obtained from NS0 cells containing three vector copies per cell (160). Other factors than the number of gene copies play an important role to achieve high production levels of antibodies. Therefore, industrial antibody expression platforms employ efficient screening systems in order to isolate the best of the high producers. However, there are also strategies to facilitate the isolation of high producer clones (161). To overcome negative effects of the integration site, protective cis-regulatory elements include insulators, boundary elements, scaffold/matrix attachment regions (S/MARs) (162), chromatin opening elements (163), and antirepressor elements (164) were introduced into the vector which reduced the influence of heterochromatin and stabilize transgene expression (165, 166). Silencing can be blocked by inhibition of histone deacetylation using butyrate (167) which could enhance the protein expression levels of the cells (168) but can also induce apoptosis.

Recombination enzymes, like bacteriophage P1 Cre recombinase, lambda phage integrase, or yeast Flp recombinase can efficiently catalyze the site specific integration into defined chromosomal recombination exchange cassettes which have been introduced into producer cell lines (169, 170). The 2A/furin technology allows expression of both IgG chains as a single gene due to post-translational auto-cleavage of the viral protease 2A encoded by the linker and subsequent processing by the Golgi protease furin (171, 172).

#### Transient production of antibodies in mammalian cells

The generation of high producer cell lines has been dramatically improved and accelerated (161, 173), however it is still too expensive, time-consuming and laborious for research applications, or if large numbers of individual antibodies have to be produced. Here, transient and semi-stable mammalian antibody expression is much more suitable because it allows fast and parallelized production without any need to generate producer cell lines (174). Moreover, transient mammalian antibody production can be scaled up by employing batch or fed-batch bioreactor processes to more than 150 L production volumes (175). Therefore, transient antibody production is suitable for small-scale production in antibody screening (176), but also capable to generate grams of antibodies (177–179).

The human embryonic kidney (HEK) 293 cell lines have been widely used for transient protein expression because they can be very efficiently transfected with plasmid DNA. Some derivatives were further transformed either with the simian virus 40 (SV40) large T antigen, termed HEK293T, or with the Epstein Barr virus (EBV) nuclear antigen 1 (EBNA1), termed HEK293E, in order to mediate semi-stable episomal propagation of vectors containing an origin of replication (ori) of SV40 or EBV, respectively. Transient transfection of plasmid DNA in HEK293 cells can also be performed in large scale by calcium phosphate transfection (180), cationic liposomes, and polymers like polyethyleneimine (PEI) (181, 182).

Recently, transient production of IgG-like scFv-Fc antibodies in the HEK293-6E cell line, a genetically modified variant with a truncated version of EBNA1 growing in suspension and chemically defined serum-free medium (183, 184), achieved volumetric yields of up to 0.6 g/L by simple shake flask cultivation. Improved production media, fed-batch supplementation, and well-controlled bioreactor processes allow higher cell densities and prolonged production time, both enhancing the yield. Backliwal and colleagues (177) combined optimized PEI-based transfection at high-cell densities with the coexpression of cell cycle regulators p18 and p21, acidic fibroblast growth factor, valproic acid supplementation, consequent maintenance at high-cell densities of cells/milliliter and up-scaling to 2 L and achieved production levels of more than 1 g IgG within 2 weeks after transient transfection.

## Transgenic Organisms

### Transgenic plants

The development of transgenic plants for the expression of recombinant antibodies is becoming interesting, especially when high amounts are required. Up-scaling of this production system can be achieved more easily compared to other systems such as mammalian cell culture, where up-scaling of the fermentation process leads to increasing production costs. In theory, the costs of an IgA expressed in plants are only 1–10% compared to the expression in hybridoma cells (185).

The generation of genetically modified dicotyledonous plants is mainly done by the transfer of the expression cassette of the transgene with the help of *Agrobacterium tumefaciens*. In principle, the gene of interest is cloned into the T-DNA of a binary plasmid (186, 187) which is flanked by two 25 bp imperfect repeats. In most cases, the expression of the transgene is under the control of one or two (188) copies of the constitutive cauliflower mosaic virus (CaMV). In addition, a selection marker is located on the T-DNA and transferred into the host genome for effective screening of successfully transformed plants. After integration of the T-DNA into the host genome by non-homologous recombination complete plants can be regenerated from transformed pieces of the plant [RB (189)]. As this procedure requires several months of transformation and special regeneration protocols, transient expression systems have been developed which allow time saving production of recombinant proteins: McCormick and colleagues designed a tobacco mosaic virus (TMV) based vector for the secretory expression of different scFvs for the treatment of non-Hodgkin’s lymphoma (190). Expression yields in *Nicotiana benthamiana* were up to 100–800 μg/mL in the crude secretory extract. Same technique has been applied for the expression of idiotype-scFvs for personalized vaccination of follicular B-cell lymphoma patients in a phase I clinical study (191). In this study, nearly half of the treated patients developed an antigen specific immune response despite differences in glycosylation pattern.

Differences in the glycosylation pattern between mammalia and plants are one of the main obstacles researchers have to overcome when developing therapeutic antibodies expressed in plants. Although plants are able to perform complex glycosylation, differences in glycosylation patterns, in particular β1,2-xylose and α1,3-fucose, can lead to immunogenicity of the therapeutic proteins (192–194). Therefore, different strategies have been developed to express recombinant proteins with a more mammalian-like glycosylation pattern. The first one is the retention of the protein in the endoplasmic reticulum (eR) as eR-associated N-glycosylation leads to the generation of oligomannose-type *N*-glycans which are identical in plants and mammalians (192, 195). One side effect of this localization is the accumulation to higher levels in the eR (196, 197). A second approach for the expression of proteins with mammalian-like glycosylation patterns is the usage of glyco-engineered plants. In most cases, RNA interference (RNAi) is used for the down-regulation of endogenous beta1,2-xylosyltransferase and alpha1,3-fucosyltransferase leading to a reduction of the xylosylated and core-fucosylated *N*-glycans (198–200). A second type of glyco-engineering in plants is the coexpression of genes which facilitates the expression of human-like *N*-glycans (201) or even the *in planta* protein sialylation by the coexpression of six mammalian genes (202). An increasing effort has been put into the adaptation of N-glycosylation, but there are also some efforts in the engineering of sialylated mucin-type *O*-glycans to achieve the most human-like glycosylation patterns (203, 204). Alternatively, non-glycosylated antibodies which mediate protection against an inhalation anthrax spore challenge in non-human primates showed an improvement of the half-life in serum (205). Rodriguez and colleagues showed that the aglycosylated form of Nimotuzumab (currently in a phase II clinical study in the USA and Canada) produced in tobacco shares the *in vitro* and *in vivo* properties as well as the antitumor effect in nude mice with the glycosylated form (206).

Transient expression of an antibody in plants can be achieved using viral vectors. The main problem with this approach is the low infectivity with these vectors. Therefore, the more efficient transfer of *A. tumefaciens* was combined with the speed and high expression rate of plant RNA viruses (207). This system has been used for the expression of monoclonal antibodies in *Nicotiana benthamiana* with yields up to 0.5 g/kg fresh weight (208).

In principle, most plantibodies are expressed in tobacco (*N. tabacum* or *N. benthamiana*), but there are also production systems in *Lemna minor* (duckweed) (209–211), rice cell culture (212), *Arabidopsis thaliana* seeds (213, 214), *Medicago sativa* (alfalfa) (215), lettuce (216), and maize (217). HIV-1 neutralizing antibody 2G12 was expressed in the endosperm of maize and showed similar or even better neutralizing properties as its CHO-derived counterpart (218).

Besides the transfection or transformation of whole plants or at least organs, monoclonal BY-2 tobacco cell lines that grow in suspension have been developed (219). Flow cytometric analysis has been used to enrich cells expressing a fluorescent marker which was located on the same T-DNA with the antibody gene. Using this method for the enrichment of high expressing cells, production could be increased up to 13-fold and was shown to be stable for 10–12 months.

Much effort has been set into the establishment and development of plants producing antibodies for therapy, but so far none of these products has appeared on the market, despite of the estimated dramatic reduction of production costs (220). Nevertheless, at least two plant derived antibodies have been used in clinical trials: CaroRX was developed by Planet Biotechnology (Hayward, CA, USA) and is expressed in transgenic tobacco (221). This antibody binds to the streptococcal antigen I/II of *S. mutans*, the major causative agent of bacterial tooth decay and prevents the attachment of *S. mutans* to tooth enamel. CaroRX has entered clinical phase II (222, 223). A second plant-made anti-idiotype antibody against non-Hodgkin-lymphoma (NHL) which was successfully tested in clinical phase I study has been mentioned above (191, 224).

### Transgenic animals

In recent years the idea of expressing human antibodies in transgenic animals has increased. On the one hand the humanization of antibodies for therapeutics derived from hybridoma technology is still a laborious and time-consuming procedure which often requires the generation and characterization of a set of different humanized versions of the antibody. On the other hand the mouse or rat derived antibodies may elicit an immune response in patients (225, 226). One method for the generation of fully human binders is the antibody phage display technology (21).

Beside this, several researchers developed transgenic animals for the production and expression of human monoclonal and polyclonal antibodies: therefore, human antibodies have mostly been expressed in the milk of transgenic mice (227–230), goats (231), or even in eggs of transgenic chickens (232).

The first step toward the generation of human antibodies in animals by immunization was the transfer of a human minilocus containing unrearranged immunoglobulin variable, diversity, and joining elements linked to a human μ-chain into mice (233). In this study, approximately 4% of the extracted B-lymphocytes expressed human antibodies. The immunization of larger animals containing human chromosomal immunoglobulin loci would enable the production of even larger amounts of antibodies. Therefore, transgenic cattle were developed by the transfer of a human artificial chromosome vector containing the entire unrearranged sequences of the human immunoglobulin heavy and lambda light chain loci (234). For the improvement of the human antibody proportion and for safety reasons regarding the potential risk of BSE, the bovine immunoglobulin μ heavy chain locus and the bovine prion protein have been knocked out (235, 236). Finally, transgenic cattle carrying human immunoglobulin heavy and kappa-light chain loci have been used for immunization with anthrax protective antigen. The resulting polyclonal antibody mixture consisted of entirely human and chimeric immunoglobulins that showed high activity and were protective in an *in vivo* mouse challenge models (237). Rabbits and cattle were used for expression of a bispecific scFv targeting the melanoma-associated proteoglycan and the human CD28 molecule on T cells (238). The usage of different animals as a source for the generation of human polyclonal sera has already been initiated: the immunoglobulin gene loci have been knocked out in livestock such as pigs or rabbits (239–241). For a review of approaches for the generation of transgenic animals expressing polyclonal human antibodies see Houdebine (242).

Much of the energy in transgenic production has been set on the development of humanized mice or rats. The well established property of generating hybridoma cells from these species facilitates a streamlined approach for the generation of a cell line which stably expresses monoclonal antibodies (17, 243, 244). Using humanized mice, an anti-HIV-1 gp140 antibody was identified, but in contrast the low number of antigen specific hybridomas occurring during the generation of the clones has been observed (245). Therefore, the usage of humanized rats has been suggested to circumvent these problems and first antibodies have been developed with sub-nanomolar affinities using the so called OmniRat (246).

## Concluding Remarks

Today, mammalian cell lines represent the most widely used expression system for the production of recombinant antibodies. Several other hosts are being developed which are even able to produce antibodies with human-like glycosylation patterns. In addition to this, there are several applications where the glycosylation pattern does not play a critical role, such as for *in vitro* diagnostics or in research. Therefore, bacteria, yeasts, filamentous fungi, and insect cells can be employed in order to lower the production costs of these products. In principle, transgenic plants and animals have the highest potential for up-scaling processes to theoretically unlimited production amounts. An overview of recombinant antibodies produced in different hosts is shown in Table 1. There, however, it must be discriminated between the yield of functional antibodies after purification and the total yield.

**Table 1:** **Production of recombinant antibodies by host**.

Host	Antigen	Antibody format (clone)	Production system	Yield	Reference
**GRAM-NEGATIVE BACTERIA**					
*Escherichia coli*	Digoxin	Fab (26–10)	Shake flask	0.8 mg/L/OD_600_	Levy et al. (49)
*Escherichia coli*	CD18	F (ab′)_2_	Fermentor	2.5 g/L	Chen et al. (247 )
*Escherichia coli*	Lysozyme	scFv (D1.3)	250/400 mL shake flask	0.3–1.0 mg/L	Jordan et al. (84), Monsellier and Bedouelle (248), Thie et al. (70)
*Escherichia coli*	CRP	scFv (LA13-IIE3)	300 mL shake flask	0.55 mg/L	Jordan et al. (83)
*Escherichia coli*	Lysozyme	scFab (D1.3)	300 mL shake flask	9.5 μg/L	
*Escherichia coli*	MUC1	VHH	100 L shake flask	10 mg/L	Rahbarizadeh et al. (249)
*Escherichia coli*	*Clostridium difficile* toxin A	VHH (14 different)	Shake flask?	1.2–72.3 mg/L	Hussack et al. (250)
*Escherichia coli*	MUC1	scFv (2 different)	250 mL shake flask	0.46/1.3 mg/L	Thie et al. (70)
*Escherichia coli*	p815^HER2^	Fab	10 L fermenter	1–2 g/L	Carter et al. (251 )
*Escherichia coli*	Atrazine	Fab (K411B)	2 L fermenter	13.8 mg/L	Wiebe et al. (66)
*Escherichia coli*	PPL	VL dAb	1.5 L fermenter	35–65 mg/L	Cossins et al. (252)
*Escherichia coli*	phOx	scFv	50 mL shake flask	16.2 mg/L	Kipriyanov et al. (69)
*Escherichia coli*	phOx	scFv	3 L fermenter	1.2 g/L	Sletta et al. (68)
*Escherichia coli*	Scorpion toxin Cn2	scFv; Fab (BCF2)	n. d.	0.3 mg/L; 1.0 mg/L	Quintero-Hernández et al. (253)
*Escherichia coli*	TNF alpha	scFv	Shake flask?	45 mg/L	Yang et al. (254)
*Escherichia coli*	HSP70	Fab (cmHsp70.1)	8 L fermenter	>15 mg/L	Friedrich et al. (64 )
*Escherichia coli*	Tissue factor	IgG	10 L fermenter	130–150 mg/L	Simmons et al. (73)
*Escherichia coli*	TAG-72	Fv (B72.3)	Shake flask; fermentor	40 mg/L; 450 mg/L	King et al. (255)
*Escherichia coli*	VEGF	scFv::SUMO	50 mL shake flask?	50.3 mg/L	Ye et al. (256)
*Escherichia coli*	HIV capsid	Fab, engineered	Shake flask	12 mg/L	Nadkarni et al. (257)
*Escherichia coli*	Ovarian carcinoma/CD3	scFv–scFv	250 mL shake flask	1.2 g/L	Zhao et al. (71)
*Escherichia coli*	Fibroblast growth factor receptor FGFR1	VHH	Shake flask	10–15 mg/L	Veggiani and de Marco (50)
*Escherichia coli*	Human prion	scFv	Shake flask	35 mg/L	Padiolleau-Lefevre et al. (258)
*Escherichia coli*	Lysozyme	scFv (D1.3)	LEX bioreactor (1.5 L)	∼2 mg/L	Miethe et al. (67 )
*Escherichia coli*	MUC1	scFv (HT186-D11)	LEX bioreactor (1.5 L)	∼40 mg/L	
*Escherichia coli*	CD30	scFv (SH313-B5)	LEX bioreactor (1.5 L)	∼38 mg/L	
*Escherichia coli*	Crf2	scFv (MS112-IIB1)	LEX bioreactor (1.5 L)	∼4.5 mg/L	
*Escherichia coli*	Tubulin	scFv (different ones)	Shake flask (intracellular)	up to 50 mg/L	Philibert et al. (259)
*Escherichia coli*	n. d.	Fab	20 L; 75 L fed-batch bioreactor	0.7 g/L; 0.5 g/L	Nesbeth et al. (260)
*Proteus mirabilis*	FAP	scFv (OS4)	50 mL shake flask	∼12 mg/L	Rippmann et al. (74 )
*Proteus mirabilis*	Phosphorylcholine	scFv-dHLX	n. d.	10–18 mg/L	Kujau et al. (30)
*Pseudomonas putidas*	Lysozyme	scFv (D1.3)	200 mL shake flask	1.5 mg/L	Dammeyer et al. (75)
*Pseudomonas putidas*	MUC1	scFv (HT186-D11)	200 mL shake flask	3.6 mg/L	
*Pseudomonas putidas*	CRP	scFv (TOB5-D4)	200 mL shake flask	2.9 mg/L	
**GRAM-POSITIVE BACTERIA**					
*Bacillus brevis*	uPA	Fab	2 L shake flask	100 mg/L	Inoue et al. (76)
*Bacillus megaterium*	Lysozyme	scFv (D1.3)	400 mL shake flask	0.41 mg/L	Jordan et al. (84)
*Bacillus megaterium*	CRP	scFv (LA13-IIE3)	300 mL shake flask	0.39 mg/L	Jordan et al. (83)
*Bacillus megaterium*	Lysozyme	scFab (D1.3)	300 mL shake flask	3.5 μg/L	Jordan et al. (83 )
*Bacillus subtilis*	Digoxin	scFv	n. d.	12 mg/L	Wu et al. (78)
*Lactobacillus paracasei*	Rotavirus	VHH	n. d.	∼1 mg/L	Pant et al. (35 )
*Streptomyces lividans*	Lysozyme	Fv	n. d.	∼1 mg/L	Ueda et al. (261 )
**EUKARYOTES**					
**Yeast**
*Yarrowia lipolytica*, *Kluyveromyces lactis*	Ras	scFv	Shake flasks	10–20 mg/L	Swennen et al. (262)
*Pichia pastoris*	Muc1	VHH	Baffled flasks	10–15 mg/L	Rahbarizadeh et al. (263)
*Pichia pastoris*	TNFα	VHH-Fc	Shake flasks	5 mg/L	Ji et al. (264 )
*Pichia pastoris*	AaHI	VHH	Shake flasks	17 mg/L	Ezzine et al. (265)
*Pichia pastoris*	B-type natriuretic peptide	scFv	Shake flasks	150 mg/L	Maeng et al. (266)
*Pichia pastoris*	Atrazine	Fab-HRP	Shake flasks	3–10 mg/L	Koliasnikov et al. (267)
*Pichia pastoris*	Muc1	Bibody, tribody	Shake flasks	12–36 mg/L	Schoonooghe et al. (268)
*Saccharomyces cerevisiae*	71 Different	VHH	Shake flasks	<1 to>100 mg/L	Gorlani et al. (269, 270)
*Pichia pastoris*	HER2	scFv	Shake flasks	15–20 mg/L	Sommaruga et al. (271)
*Pichia pastoris*	n. d.	scFv	n. d.	300 mg/L	Khatri et al. (272)
*Pichia pastoris*	Keratin 8	sc (Fv)_2_	Baffled shake flasks	4–5 mg/L	Jafari et al. (273)
*Pichia pastoris*	n. d.	IgG	0.5 L bioreactor	0.5–1 g/L	Barnard et al. (274)
*Pichia pastoris*	Rabies virus	scFv-Fc	80 L fermenter	60 mg/L	Wang et al. (275)
*Pichia pastoris*	HER2	IgG	3 L bioreactor	148–227 mg/L	Chen et al. (276)
**Filamentous fungi**
*Aspergillus niger* var. *awamori*	ErbB2	IgG, Fab	Shake flasks	0.9 g/L; 0.2 g/L	Ward et al. (118)
*Aspergillus niger* var. *awamori*	Lysozyme	scFv	7 L fermenter	108.9 mg/L	Sotiriadis et al. (277)
*Aspergillus oryzae*	EGFR	VHH	Shake flasks	73.8 mg/L	Okazaki et al. (123)
**Protozoa**
*Leishmania tarentolae*	16 Different scFv	scFv	Shake flasks	0.04–3.38 mg/L	Klatt and Konthur (128)
**Insect cells**
*Trichoplusia ni* larvae	Rotavirus	VHH	Living larvae	257 mg/L	Gómez-Sebastián et al. (278)
*Spodoptera frugiperda*	Blood coagulation factor VIII	scFv	Shake flasks	3.2–10 mg/L	Kurasawa et al. (279)
*Drosophila*, S2	Glycoprotein H	Fab	Spinner flasks	16 mg/L	Backovic et al. (280)
*Drosophila*, S2	HIV	IgG	n. d.	5–35 mg/L	Johansson et al. (281)
*Drosophila*, S2	Bovine viral diarrhea virus, hepatitis C virus	scFv	n. d.	5–12 mg/L	Gilmartin et al. (282)
*Spodoptera frugiperda*, SF-9	gp41	IgG	T-flasks	3 mg/L	Palmberger et al. (283)
*Trichoplusia ni*	gp41	IgG	T-flasks	12 mg/L	Palmberger et al. (283)
*Sf* SWT-1 Mimic	gp41	IgG	T-flasks	3 mg/L	Palmberger et al. (283)
**MAMMALIAN CELLS**					
**Transient**
*HEK293T*	CD200, SIRPγ	Fab	Genejuice, roller bottles	4 mg/L	Nettleship et al. (284 )
*HEK293T*	n. d.	IgG	HEKfectin, tissue culture plates	1–14 mg/L	Li et al. (155)
*CHO*	n. d.	IgG1, IgG4	Lipofectamine	140 mg/L	Codamo et al. (285)
*HEK293F*	n. d.	IgG	293fectin	100–400 mg/L	Van Berkel et al. (286)
*CHO DG44*	RhD	IgG	PEI, square-shaped bottles	90 mg/L	Wulhfard et al. (287)
*HEK293E*	n. d.	IgG	PEI, square-shaped bottles	200 mg/L	Backliwal et al. (288)
*CHO*	n. d.	IgG	PEI, square-shaped bottles	60–80 mg/L	Wulhfard et al. (289)
*HEK293E*	RhD	IgG	PEI, square-shaped bottles	1.1 g/L	Backliwal et al. (177)
**Stable**
*CHO-K1*	n. d.	IgG	Lipofectamine	0.05–0.45 mg/L	Li et al. (155)
*CHO*	HIV-1	scFv-Fc	PEI	5.78–45.49 mg/L	Mader et al. (290)
*CHO*	n. d.	IgG	Electroporation	4 g/L	Kober et al. (291)
*NS0*	n. d.	IgG	5 L bioreactor, fed-batch	800 mg/L	Spens and Häggström (292)
*NS0*	n. d.	IgG	2–100 L bioreactor	2.64 g/L	Burky et al. (293)
*per.C6*	n. d.	IgG	Hollow fiber bioreactor	1 g/L	Jones et al. (147)
*per.C6*	n. d.	IgG	Roller bottle culture	50–100 mg/L	Jones et al. (147)
*per.C6*	n. d.	IgM	Shake flasks	0.5–2 g/L	Tchoudakova et al. (294)
*CHO*	n. d.	VHH-Fc	Shake flasks	100 mg/L	Agrawal et al. (295)
*CHO*	HER2	IgG	Orbital shaking bioreactor	152 mg/L	Huang et al. (296)
**Transgenic plants**
*Chlamydomonas reinhardtii*	CD22	Immunotoxin, exotoxin A	Particle bombardement	0.2–0.4% of chloroplasts	Tran et al. (297)
*Nicotiana tabacum*	BoNT/A	scFv	*Agrobacterium tumefaciens*	20–40 mg/kg	Almquist et al. (298)
*Nicotiana benthamiana*	HIV	IgG	CPMV	105.1 mg/kg	Sainsbury et al. (299)
*Nicotiana tabacum*	HCC	scFv-RNase	*Agrobacterium tumefaciens*	0.75–1.99 μg/g	Cui et al. (300 )
*Nicotiana benthamiana*	Ebola virus GP1	IgG	*Agrobacterium tumefaciens*	0.4–0.5 mg/g	Huang et al. (301)
**Transgenic animals**
Mouse	HBV	IgG	Milk	17.8 mg/mL	Zhang et al. (302)
Mouse	CD147	Chimeric IgG	Milk	1.1–7.4 mg/mL	Wei et al. (303)
Mouse	Hepatitis A virus	IgG	Milk	32 mg/mL	Zhang et al. (304)
Mouse	HER2	scFv-Fc	Milk	∼120 ng/mL	Yuskevich et al. (305 )
Chicken	CD2, prion peptide	Chimeric IgG	Egg white	<150 μg/mL	Kamihira et al. (306)

*AaHI, toxin class I of Androctonus australis hector scorpion venom; BoNT/A, Bolutinum toxin serotype A; CPMV, cowpea mosaic virus; FAP, fibroblast activation protein alpha; HCC, hepatocellular carcinoma; phOx, 2-phenyl-oxazoline-5on; PEI, polyethyleneimine; PPL, peptostreptococcal protein L; RhD, rhesus factor D; uPA, urokinase type plasminogen activator*.

Antibody phage display is now a widespread method for the development of antibody fragments such as scFv or Fab. The expression host used in this technology is *E. coli* which is known to be the best genetically examined organism providing a large set of molecular biological tools for genetical engineering. Consequently, both antibody generation and production can be performed without changing the production system. Using high-cell density fermentation, the yield can be up to 1–2 g/L depending on the individual antibody fragment. Antibody fragments expressed in *E. coli* are mainly secreted into the periplasm and have to be extracted from there. Gram-positive bacteria lack the outer membrane and are well suited for biotechnological processes due to their powerful secretion apparatus which allow easy purification directly from the cultivation supernatant. However, antibody production systems employing Gram-positive bacteria are still in the developmental stage. However, larger antibody formats are very difficult to express in bacteria, if they can be expressed at all. Furthermore, the lack of a glycosylation apparatus limits their use, if effector functions are needed.

Yeasts, as an eukaryotic organism, has the capacity to perform post-translational modifications. In addition, they can be used even in high throughput processes and glyco-engineering enables the expression of recombinant proteins with human-like glycosylation. Nevertheless, the production of full-size immunoglobulins remains a challenge. Compared to yeasts, filamentous fungi are more difficult for the generation of transformed clones, but they have a long tradition for the usage in biotechnology and they have partially been used for the expression of IgGs. In contrast, the development of protozoa as an expression system for recombinant proteins and antibodies has just been started and is still in a developmental stage. However, the mammalian-like glycosylation pattern presents them a promising candidate for further exploitation.

Insect cells contain a better suited protein folding and secretion apparatus than prokaryotes. Their high robustness combined with less sophisticated requirements for fermentation provide some advantages compared to mammalian cells. However, the development of stable insect cell lines and process technology is not developed as far. Consequently, mammalian cell lines are most widely used for the production of therapeutic antibodies as they provide a sophisticated folding and secretion apparatus as well as human-like glycosylation. For the production of high levels of recombinant antibodies high technical efforts are needed leading to relatively high costs. The maximum reported yield of functional IgG was 5 g/L which cannot be achieved using other expression systems so far, but up-scaling of the production does not lead to a high reduction of the production costs.

For an efficient reduction of production costs, transgenic plants can be used as they represent a highly scalable expression system; cultivation can be easily expanded without a gross increase in costs. In contrast, the generation of transgenic plants remains very complex and difficult. The most important obstacle of transgenic plants is the downstream processing as tons of plant material may have to be processed. However, antibody production in milk or eggs of animals would also be highly scalable and permits easy downstream processing. Several livestock animal species have been developed for the expression of recombinant proteins, but generation of transgenic animals also is very laborious. An interesting approach is the combination of human transgenic animals with hybridoma technology for the development of human antibodies.

In principle, there is no “universal” production system which can guarantee high yields of recombinant antibody, particularly as every antibody-based molecule itself will cause its own issues in terms of expression.

## Conflict of Interest Statement

The authors declare that the research was conducted in the absence of any commercial or financial relationships that could be construed as a potential conflict of interest.

## Abbreviations

IgG, immunoglobulin G; mAb, monoclonal antibody.

## References

[1] Colwill K Renewable Protein Binder Working Group Gräslund S A roadmap to generate renewable protein binders to the human proteome. Nat Methods (2011) 8:551–8 10.1038/nmeth.1607 21572409

[2] Mehan MR Ostroff R Wilcox SK Steele F Schneider D Jarvis TC Highly multiplexed proteomic platform for biomarker discovery, diagnostics, and therapeutics. Adv Exp Med Biol (2013) 735:283–300 23402035 10.1007/978-1-4614-4118-2_20

[3] Foudeh AM Fatanat Didar T Veres T Tabrizian M Microfluidic designs and techniques using lab-on-a-chip devices for pathogen detection for point-of-care diagnostics. Lab Chip (2012) 12:3249–66 10.1039/c2lc40630f 22859057

[4] Uttamchandani M Neo JL Ong BNZ Moochhala S Applications of microarrays in pathogen detection and biodefence. Trends Biotechnol (2009) 27:53–61 10.1016/j.tibtech.2008.09.004 19008003 PMC7114317

[5] Van Breedam W Costers S Vanhee M Gagnon CA Rodríguez-Gómez IM Geldhof M Porcine reproductive and respiratory syndrome virus (PRRSV)-specific mAbs: supporting diagnostics and providing new insights into the antigenic properties of the virus. Vet Immunol Immunopathol (2011) 141:246–57 10.1016/j.vetimm.2011.03.008 21470695

[6] Van Hoeven KH Dale C Foster P Body B Comparison of three enzyme-linked immunosorbent assays for detection of immunoglobulin g antibodies to tetanus toxoid with reference standards and the impact on clinical practice. Clin Vaccine Immunol (2008) 15:1751–4 10.1128/CVI.00254-08 18845832 PMC2593177

[7] Zasada AA Rastawicki W Smietanska K Rokosz N Jagielski M Comparison of seven commercial enzyme-linked immunosorbent assays for the detection of anti-diphtheria toxin antibodies. Eur J Clin Microbiol Infect Dis (2013) 32:891–7 10.1007/s10096-013-1823-y 23354678

[8] Dübel S editor. Handbook of Therapeutic Antibodies. Weinheim: Wiley-VCH (2008). 10.1002/9783527619740

[9] Reichert JM Which are the antibodies to watch in 2013? MAbs (2013) 5:1–4 10.4161/mabs.22976 23254906 PMC3564874

[10] Eisenberg SA Biologic therapy. J Infus Nurs (2012) 35:301–13 10.1097/NAN.0b013e31826579aa 22955152

[11] Dimitrov DS Therapeutic proteins. In: Voynov V Caravella JA editors. Therapeutic Proteins, Methods in Molecular Biology. Clifton, NJ: Humana Press (2012). p. 1–26 10.1007/978-1-61779-921-1_1 PMC6988726 22735943

[12] Wilde H Thipkong P Sitprija V Chaiyabutr N Heterologous antisera and antivenins are essential biologicals: perspectives on a worldwide crisis. Ann Intern Med (1996) 125:233–6 10.7326/0003-4819-125-3-199608010-00012 8686982

[13] Köhler G Milstein C Continuous cultures of fused cells secreting antibody of predefined specificity. Nature (1975) 256:495–7 10.1038/256495a0 1172191

[14] Reichert JM Antibody-based therapeutics to watch in 2011. MAbs (2011) 3:76–99 10.4161/mabs.3.1.13895 21051951 PMC3038014

[15] Reichert JM Antibodies to watch in 2010. MAbs (2010) 2:84–100 10.4161/mabs.2.1.10677 20065640 PMC2828582

[16] Nemazee DA Bürki K Clonal deletion of B lymphocytes in a transgenic mouse bearing anti-MHC class I antibody genes. Nature (1989) 337:562–6 10.1038/337562a0 2783762

[17] Taylor LD Carmack CE Schramm SR Mashayekh R Higgins KM Kuo CC A transgenic mouse that expresses a diversity of human sequence heavy and light chain immunoglobulins. Nucleic Acids Res (1992) 20:6287–95 10.1093/nar/20.23.6287 1475190 PMC334518

[18] Breitling F Dübel S Seehaus T Klewinghaus I Little M A surface expression vector for antibody screening. Gene (1991) 104:147–53 10.1016/0378-1119(91)90244-6 1916287

[19] Edwards BM He M Evolution of antibodies in vitro by ribosome display. Methods Mol Biol (2012) 907:281–92 22907358 10.1007/978-1-61779-974-7_16

[20] Hoogenboom HR Selecting and screening recombinant antibody libraries. Nat Biotechnol (2005) 23:1105–16 10.1038/nbt1126 16151404

[21] Schirrmann T Meyer T Schütte M Frenzel A Hust M Phage display for the generation of antibodies for proteome research, diagnostics and therapy. Molecules (2011) 16:412–26 10.3390/molecules16010412 21221060 PMC6259421

[22] Thom G Groves M Ribosome display. Methods Mol Biol (2012) 901:101–16 10.1007/978-1-61779-931-0_6 22723096

[23] Edelman GM Antibody structure and molecular immunology. Science (1973) 180:830–40 10.1126/science.180.4088.830 4540988

[24] Bird RE Hardman KD Jacobson JW Johnson S Kaufman BM Lee SM Single-chain antigen-binding proteins. Science (1988) 242:423–6 10.1126/science.3140379 3140379

[25] Schmiedl A Breitling F Winter CH Queitsch I Dübel S Effects of unpaired cysteines on yield, solubility and activity of different recombinant antibody constructs expressed in E. coli. J Immunol Methods (2000) 242:101–14 10.1016/S0022-1759(00)00243-X 10986393

[26] Hust M Jostock T Menzel C Voedisch B Mohr A Brenneis M Single chain Fab (scFab) fragment. BMC Biotechnol (2007) 7:14 10.1186/1472-6750-7-14 17346344 PMC1829395

[27] Hudson PJ Kortt AA High avidity scFv multimers; diabodies and triabodies. J Immunol Methods (1999) 231:177–89 10.1016/S0022-1759(99)00157-X 10648937

[28] Hu S Shively L Raubitschek A Sherman M Williams LE Wong JY Minibody: a novel engineered anti-carcinoembryonic antigen antibody fragment (single-chain Fv-CH3) which exhibits rapid, high-level targeting of xenografts. Cancer Res (1996) 56:3055–61 8674062

[29] Kipriyanov SM Breitling F Little M Dübel S Single-chain antibody streptavidin fusions: tetrameric bifunctional scFv-complexes with biotin binding activity and enhanced affinity to antigen. Hum Antibodies Hybridomas (1995) 6:93–101 8597629

[30] Kujau MJ Hoischen C Riesenberg D Gumpert J Expression and secretion of functional miniantibodies McPC603scFvDhlx in cell-wall-less L-form strains of *Proteus mirabilis a*nd *Escherichia coli*: a comparison of the synthesis capacities of L-form strains with an *E. coli* producer strain. Appl Microbiol Biotechnol (1998) 49:51–8 10.1007/s002530051136 9487710

[31] Plückthun A Pack P New protein engineering approaches to multivalent and bispecific antibody fragments. Immunotechnology (1997) 3:83–105 10.1016/S1380-2933(97)00067-5 9237094

[32] Choi BD Kuan C-T Cai M Archer GE Mitchell DA Gedeon PC Systemic administration of a bispecific antibody targeting EGFRvIII successfully treats intracerebral glioma. Proc Natl Acad Sci U S A (2013) 110:270–5 10.1073/pnas.1219817110 23248284 PMC3538214

[33] Fournier P Schirrmacher V Bispecific antibodies and trispecific immunocytokines for targeting the immune system against cancer: preparing for the future. BioDrugs (2013) 27:35–53 10.1007/s40259-012-0008-z 23329400

[34] Zitron IM Thakur A Norkina O Barger GR Lum LG Mittal S Targeting and killing of glioblastoma with activated T cells armed with bispecific antibodies. BMC Cancer (2013) 13:83 10.1186/1471-2407-13-83 23433400 PMC3599512

[35] Pant N Hultberg A Zhao Y Svensson L Pan-Hammarstrom Q Johansen K Lactobacilli expressing variable domain of llama heavy-chain antibody fragments (lactobodies) confer protection against rotavirus-induced diarrhea. J Infect Dis (2006) 194:1580–8 10.1086/508747 17083044

[36] Holt LJ Herring C Jespers LS Woolven BP Tomlinson IM Domain antibodies: proteins for therapy. Trends Biotechnol (2003) 21:484–90 10.1016/j.tibtech.2003.08.007 14573361

[37] Tang Z Feng M Gao W Phung Y Chen W Chaudhary A A human single-domain antibody elicits potent anti-tumor activity by targeting an epitope in mesothelin close to the cancer cell surface. Mol Cancer Ther (2013) 12:416–26 10.1158/1535-7163.MCT-12-0731 23371858 PMC3624043

[38] Powers DB Amersdorfer P Poul M Nielsen UB Shalaby MR Adams GP Expression of single-chain Fv-Fc fusions in *Pichia pastoris*. J Immunol Methods (2001) 251:123–35 10.1016/S0022-1759(00)00290-8 11292488

[39] Wan L Zhu S Zhu J Yang H Li S Li Y Production and characterization of a CD25-specific scFv-Fc antibody secreted from *Pichia pastoris*. Appl Microbiol Biotechnol (2012) 97:3855–63 10.1007/s00253-012-4632-9 23250227

[40] Ni Y Chen R Extracellular recombinant protein production from *Escherichia coli*. Biotechnol Lett (2009) 31:1661–70 10.1007/s10529-009-0077-3 19597765

[41] Schmidt FR Recombinant expression systems in the pharmaceutical industry. Appl Microbiol Biotechnol (2004) 65:363–72 10.1007/s00253-004-1656-9 15480623

[42] Terpe K Overview of bacterial expression systems for heterologous protein production: from molecular and biochemical fundamentals to commercial systems. Appl Microbiol Biotechnol (2006) 72:211–22 10.1007/s00253-006-0465-8 16791589

[43] Skerra A Plückthun A Assembly of a functional immunoglobulin Fv fragment in *Escherichia coli*. Science (1988) 240:1038–41 10.1126/science.3285470 3285470

[44] Better M Chang CP Robinson RR Horwitz AH *Escherichia coli* secretion of an active chimeric antibody fragment. Science (1988) 240:1041–3 10.1126/science.3285471 3285471

[45] Wörn A Auf der Maur A Escher D Honegger A Barberis A Plückthun A Correlation between in vitro stability and in vivo performance of anti-GCN4 intrabodies as cytoplasmic inhibitors. J Biol Chem (2000) 275:2795–803 10.1074/jbc.275.4.2795 10644744

[46] Martineau P Jones P Winter G Expression of an antibody fragment at high levels in the bacterial cytoplasm. J Mol Biol (1998) 280:117–27 10.1006/jmbi.1998.1840 9653035

[47] Proba K Wörn A Honegger A Plückthun A Antibody scFv fragments without disulfide bonds made by molecular evolution. J Mol Biol (1998) 275:245–53 10.1006/jmbi.1997.1457 9466907

[48] Wörn A Plückthun A Mutual stabilization of VL and VH in single-chain antibody fragments, investigated with mutants engineered for stability. Biochemistry (1998) 37:13120–7 10.1021/bi980712q 9748318

[49] Levy R Weiss R Chen G Iverson BL Georgiou G Production of correctly folded Fab antibody fragment in the cytoplasm of *Escherichia coli* trxB gor mutants via the coexpression of molecular chaperones. Protein Expr Purif (2001) 23:338–47 10.1006/prep.2001.1520 11676610

[50] Veggiani G de Marco A Improved quantitative and qualitative production of single-domain intrabodies mediated by the co-expression of Erv1p sulfhydryl oxidase. Protein Expr Purif (2011) 79:111–4 10.1016/j.pep.2011.03.005 21421053

[51] Rusch SL Kendall DA Interactions that drive Sec-dependent bacterial protein transport. Biochemistry (2007) 46:9665–73 10.1021/bi7010064 17676771 PMC2675607

[52] Ge L Knappik A Pack P Freund C Plückthun A Expressing Antibodies in Escherichia coli, in: Antibody Engineering. New York: Oxford University Press (1995). p. 229–66

[53] Sletta H Tøndervik A Hakvåg S Aune TEV Nedal A Aune R The presence of N-terminal secretion signal sequences leads to strong stimulation of the total expression levels of three tested medically important proteins during high-cell-density cultivations of *Escherichia coli*. Appl Environ Microbiol (2007) 73:906–12 10.1128/AEM.01804-06 17142370 PMC1800768

[54] Tachibana H Takekoshi M Cheng X-J Nakata Y Takeuchi T Ihara S Bacterial expression of a human monoclonal antibody-alkaline phosphatase conjugate specific for *Entamoeba histolytica*. Clin Diagn Lab Immunol (2004) 11:216–8 14715571 10.1128/CDLI.11.1.216-218.2004 PMC321342

[55] Thie H Schirrmann T Paschke M Dübel S Hust M SRP and Sec pathway leader peptides for antibody phage display and antibody fragment production in *E. coli*. N Biotechnol (2008) 25:49–54 10.1016/j.nbt.2008.01.001 18504019

[56] Kirsch M Zaman M Meier D Dübel S Hust M Parameters affecting the display of antibodies on phage. J Immunol Methods (2005) 301:173–85 10.1016/j.jim.2005.04.017 15992816

[57] Ward ES Antibody engineering using *Escherichia coli* as host. Adv Pharmacol (1993) 24:1–20 10.1016/S1054-3589(08)60931-X 8504061

[58] Hust M Steinwand M Al-Halabi L Helmsing S Schirrmann T Dübel S Improved microtitre plate production of single chain Fv fragments in *Escherichia coli*. N Biotechnol (2009) 25:424–8 10.1016/j.nbt.2009.03.004 19552889

[59] Lauer B Ottleben I Jacobsen H-J Reinard T Production of a single-chain variable fragment antibody against fumonisin B1. J Agric Food Chem (2005) 53:899–904 10.1021/jf048651s 15712995

[60] Mersmann M Meier D Mersmann J Helmsing S Nilsson P Gräslund S Towards proteome scale antibody selections using phage display. N Biotechnol (2010) 27:118–28 10.1016/j.nbt.2009.10.007 19883803

[61] Mi J Yan J Guo Z Zhao M Chang W Isolation and characterization of an anti-recombinant erythropoietin single-chain antibody fragment using a phage display antibody library. Anal Bioanal Chem (2005) 383:218–23 10.1007/s00216-005-3401-3 16158293

[62] Bothmann H Plückthun A Selection for a periplasmic factor improving phage display and functional periplasmic expression. Nat Biotechnol (1998) 16:376–80 10.1038/nbt0498-376 9555730

[63] Bothmann H Pluckthun A The periplasmic *Escherichia coli* peptidyl prolyl cis,trans-isomerase FkpA. I. Increased functional expression of antibody fragments with and without cis-prolines. J Biol Chem (2000) 275:17100–5 10.1074/jbc.M910233199 10748200

[64] Friedrich L Stangl S Hahne H Küster B Köhler P Multhoff G Bacterial production and functional characterization of the Fab fragment of the murine IgG1/lambda monoclonal antibody cmHsp70.1, a reagent for tumour diagnostics. Protein Eng Des Sel (2010) 23:161–8 10.1093/protein/gzp095 20123884

[65] Ramm K Plückthun A The periplasmic *Escherichia coli* peptidyl prolyl cis,trans-isomerase FkpA. II. Isomerase-independent chaperone activity in vitro. J Biol Chem (2000) 275:17106–13 10.1074/jbc.M910234199 10748201

[66] Wiebe JC Schüller C Reiche JA Kramer K Skerra A Hock B An expression system for the *E. coli* fermentation of recombinant antibody Fab fragments from mice and rabbits. J AOAC Int (2010) 93:80–8 20334168

[67] Miethe S Meyer T Wöhl-Bruhn S Frenzel A Schirrmann T Dübel S Production of single chain fragment variable (scFv) antibodies in *Escherichia coli* using the LEX^TM^ bioreactor. J Biotechnol (2012) 163:105–11 10.1016/j.jbiotec.2012.07.011 22902410

[68] Sletta H Nedal A Aune TEV Hellebust H Hakvåg S Aune R Broad-host-range plasmid pJB658 can be used for industrial-level production of a secreted host-toxic single-chain antibody fragment in *Escherichia coli*. Appl Environ Microbiol (2004) 70:7033–9 10.1128/AEM.70.12.7033-7039.2004 15574897 PMC535149

[69] Kipriyanov SM Moldenhauer G Little M High level production of soluble single chain antibodies in small-scale *Escherichia coli* cultures. J Immunol Methods (1997) 200:69–77 10.1016/S0022-1759(96)00188-3 9005945

[70] Thie H Binius S Schirrmann T Hust M Dübel S Multimerization domains for antibody phage display and antibody production. N Biotechnol (2009) 26:314–21 10.1016/j.nbt.2009.07.005 19631299

[71] Zhao J-B Wei D-Z Tong W-Y Identification of *Escherichia coli* host cell for high plasmid stability and improved production of antihuman ovarian carcinoma x antihuman CD3 single-chain bispecific antibody. Appl Microbiol Biotechnol (2007) 76:795–800 10.1007/s00253-007-1050-5 17598107

[72] Mazor Y Van Blarcom T Mabry R Iverson BL Georgiou G Isolation of engineered, full-length antibodies from libraries expressed in *Escherichia coli*. Nat Biotechnol (2007) 25:563–5 10.1038/nbt1296 17435747

[73] Simmons LC Reilly D Klimowski L Raju TS Meng G Sims P Expression of full-length immunoglobulins in *Escherichia coli*: rapid and efficient production of aglycosylated antibodies. J Immunol Methods (2002) 263:133–47 10.1016/S0022-1759(02)00036-4 12009210

[74] Rippmann JF Klein M Hoischen C Brocks B Rettig WJ Gumpert J Procaryotic expression of single-chain variable-fragment (scFv) antibodies: secretion in L-form cells of *Proteus mirabilis* leads to active product and overcomes the limitations of periplasmic expression in *Escherichia coli*. Appl Environ Microbiol (1998) 64:4862–9 9835575 10.1128/aem.64.12.4862-4869.1998 PMC90935

[75] Dammeyer T Steinwand M Krüger S-C Dübel S Hust M Timmis KN Efficient production of soluble recombinant single chain Fv fragments by a *Pseudomonas putida* strain KT2440 cell factory. Microb Cell Fact (2011) 10:11 10.1186/1475-2859-10-11 21338491 PMC3053225

[76] Inoue Y Ohta T Tada H Iwasa S Udaka S Yamagata H Efficient production of a functional mouse/human chimeric Fab’ against human urokinase-type plasminogen activator by *Bacillus brevis*. Appl Microbiol Biotechnol (1997) 48:487–92 10.1007/s002530051084 9390457

[77] Shiroza T Shinozaki-Kuwahara N Hayakawa M Shibata Y Hashizume T Fukushima K Production of a single-chain variable fraction capable of inhibiting the *Streptococcus mutans* glucosyltransferase in *Bacillus brevis*: construction of a chimeric shuttle plasmid secreting its gene product. Biochim Biophys Acta (2003) 1626:57–64 10.1016/S0167-4781(03)00038-1 12697330

[78] Wu SC Ye R Wu XC Ng SC Wong SL Enhanced secretory production of a single-chain antibody fragment from *Bacillus subtilis* by co-production of molecular chaperones. J Bacteriol (1998) 180:2830–5 9603868 10.1128/jb.180.11.2830-2835.1998 PMC107245

[79] Wu S-C Yeung JC Duan Y Ye R Szarka SJ Habibi HR Functional production and characterization of a fibrin-specific single-chain antibody fragment from *Bacillus subtilis*: effects of molecular chaperones and a wall-bound protease on antibody fragment production. Appl Environ Microbiol (2002) 68:3261–9 10.1128/AEM.68.7.3261-3269.2002 12089002 PMC126797

[80] David F Steinwand M Hust M Bohle K Ross A Dübel S Antibody production in *Bacillus megaterium*: strategies and physiological implications of scaling from microtiter plates to industrial bioreactors. Biotechnol J (2011) 6:1516–31 10.1002/biot.201000417 21805641

[81] David F Westphal R Bunk B Jahn D Franco-Lara E Optimization of antibody fragment production in *Bacillus megaterium*: the role of metal ions on protein secretion. J Biotechnol (2010) 150:115–24 10.1016/j.jbiotec.2010.07.023 20670661

[82] Jordan E Al-Halabi L Schirrmann T Hust M Antibody production by the Gram-positive bacterium *Bacillus megaterium*. Methods Mol Biol (2009) 525:509–16 10.1007/978-1-59745-554-1_27 19252842

[83] Jordan E Al-Halabi L Schirrmann T Hust M Dübel S Production of single chain Fab (scFab) fragments in *Bacillus megaterium*. Microb Cell Fact (2007) 6:38 10.1186/1475-2859-6-2 18042285 PMC2212634

[84] Jordan E Hust M Roth A Biedendieck R Schirrmann T Jahn D Production of recombinant antibody fragments in *Bacillus megaterium*. Microb Cell Fact (2007) 6:2 10.1186/1475-2859-6-2 17224052 PMC1797049

[85] Lüders S David F Steinwand M Jordan E Hust M Dübel S Influence of the hydromechanical stress and temperature on growth and antibody fragment production with *Bacillus megaterium*. Appl Microbiol Biotechnol (2011) 91:81–90 10.1007/s00253-011-3193-7 21479717

[86] Vary PS Prime time for *Bacillus megaterium*. Microbiology (1994) 140(Pt 5):1001–13 10.1099/13500872-140-5-1001 8025666

[87] Chancey CJ Khanna KV Seegers JFML Zhang GW Hildreth J Langan A Lactobacilli-expressed single-chain variable fragment (scFv) specific for intercellular adhesion molecule 1 (ICAM-1) blocks cell-associated HIV-1 transmission across a cervical epithelial monolayer. J Immunol (2006) 176:5627–36 16622032 10.4049/jimmunol.176.9.5627

[88] Krüger C Hu Y Pan Q Marcotte H Hultberg A Delwar D In situ delivery of passive immunity by lactobacilli producing single-chain antibodies. Nat Biotechnol (2002) 20:702–6 10.1038/nbt0702-702 12089555

[89] Marcotte H Kõll-Klais P Hultberg A Zhao Y Gmür R Mändar R Expression of single-chain antibody against RgpA protease of *Porphyromonas gingivalis* in *Lactobacillus*. J Appl Microbiol (2006) 100:256–63 10.1111/j.1365-2672.2005.02786.x 16430501

[90] De Pourcq K Vervecken W Dewerte I Valevska A Van Hecke A Callewaert N Engineering the yeast *Yarrowia lipolytica* for the production of therapeutic proteins homogeneously glycosylated with Man_8_GlcNAc_2_ and Man_5_GlcNAc_2_. Microb Cell Fact (2012) 11:53 10.1186/1475-2859-11-53 22548968 PMC3512530

[91] Schreuder MP Mooren AT Toschka HY Verrips CT Klis FM Immobilizing proteins on the surface of yeast cells. Trends Biotechnol (1996) 14:115–20 10.1016/0167-7799(96)10017-2 8936431

[92] Jeong KJ Jang SH Velmurugan N Recombinant antibodies: engineering and production in yeast and bacterial hosts. Biotechnol J (2011) 6:16–27 10.1002/biot.201000381 21170983

[93] Davis GT Bedzyk WD Voss EW Jacobs TW Single chain antibody (SCA) encoding genes: one-step construction and expression in eukaryotic cells. Biotechnology (N Y) (1991) 9:165–9 10.1038/nbt0291-165 1367186

[94] Fleer R Engineering yeast for high level expression. Curr Opin Biotechnol (1992) 3:486–96 10.1016/0958-1669(92)90076-U 1368934

[95] Buckholz RG Gleeson MA Yeast systems for the commercial production of heterologous proteins. Biotechnology (N Y) (1991) 9:1067–72 10.1038/nbt1191-1067 1367623

[96] Gasser B Maurer M Gach J Kunert R Mattanovich D Engineering of *Pichia pastoris* for improved production of antibody fragments. Biotechnol Bioeng (2006) 94:353–61 10.1002/bit.20851 16570317

[97] Emberson LM Trivett AJ Blower PJ Nicholls PJ Expression of an anti-CD33 single-chain antibody by *Pichia pastoris*. J Immunol Methods (2005) 305:135–51 10.1016/j.jim.2005.04.005 16139294

[98] Ridder R Schmitz R Legay F Gram H Generation of rabbit monoclonal antibody fragments from a combinatorial phage display library and their production in the yeast *Pichia pastoris*. Biotechnology (N Y) (1995) 13:255–60 10.1038/nbt0395-255 9634767

[99] Gurkan C Symeonides SN Ellar DJ High-level production in *Pichia pastoris* of an anti-p185HER-2 single-chain antibody fragment using an alternative secretion expression vector. Biotechnol Appl Biochem (2004) 39:115–22 10.1042/BA20030096 12962542

[100] Eldin P Pauza ME Hieda Y Lin G Murtaugh MP Pentel PR High-level secretion of two antibody single chain Fv fragments by *Pichia pastoris*. J Immunol Methods (1997) 201:67–75 10.1016/S0022-1759(96)00213-X 9032410

[101] Damasceno LM Anderson KA Ritter G Cregg JM Old LJ Batt CA Cooverexpression of chaperones for enhanced secretion of a single-chain antibody fragment in *Pichia pastoris*. Appl Microbiol Biotechnol (2007) 74:381–9 10.1007/s00253-006-0652-7 17051412

[102] Frenken LG van der Linden RH Hermans PW Bos JW Ruuls RC de Geus B Isolation of antigen specific llama VHH antibody fragments and their high level secretion by *Saccharomyces cerevisiae*. J Biotechnol (2000) 78:11–21 10.1016/S0168-1656(99)00228-X 10702907

[103] Liu J Wei D Qian F Zhou Y Wang J Ma Y pPIC9-Fc: a vector system for the production of single-chain Fv-Fc fusions in *Pichia pastoris* as detection reagents in vitro. J Biochem (2003) 134:911–7 10.1093/jb/mvg222 14769881

[104] Horwitz AH Chang CP Better M Hellstrom KE Robinson RR Secretion of functional antibody and Fab fragment from yeast cells. Proc Natl Acad Sci U S A (1988) 85:8678–82 10.1073/pnas.85.22.8678 3054890 PMC282523

[105] Potgieter TI Kersey SD Mallem MR Nylen AC d’Anjou M Antibody expression kinetics in glycoengineered *Pichia pastoris*. Biotechnol Bioeng (2010) 106:918–27 10.1002/bit.22756 20506148

[106] Van der Linden RH de Geus B Frenken GJ Peters H Verrips CT Improved production and function of llama heavy chain antibody fragments by molecular evolution. J Biotechnol (2000) 80:261–70 10.1016/S0168-1656(00)00274-1 10949316

[107] Shusta EV Raines RT Plückthun A Wittrup KD Increasing the secretory capacity of *Saccharomyces cerevisiae* for production of single-chain antibody fragments. Nat Biotechnol (1998) 16:773–7 10.1038/nbt0898-773 9702778

[108] Grinna LS Tschopp JF Size distribution and general structural features of N-linked oligosaccharides from the methylotrophic yeast, *Pichia pastoris*. Yeast (1989) 5:107–15 10.1002/yea.320050206 2711751

[109] Choi B-K Bobrowicz P Davidson RC Hamilton SR Kung DH Li H Use of combinatorial genetic libraries to humanize N-linked glycosylation in the yeast *Pichia pastoris*. Proc Natl Acad Sci U S A (2003) 100:5022–7 10.1073/pnas.0931263100 12702754 PMC154291

[110] Hamilton SR Davidson RC Sethuraman N Nett JH Jiang Y Rios S Humanization of yeast to produce complex terminally sialylated glycoproteins. Science (2006) 313:1441–3 10.1126/science.1130256 16960007

[111] Hamilton SR Bobrowicz P Bobrowicz B Davidson RC Li H Mitchell T Production of complex human glycoproteins in yeast. Science (2003) 301:1244–6 10.1126/science.1088166 12947202

[112] Li H Sethuraman N Stadheim TA Zha D Prinz B Ballew N Optimization of humanized IgGs in glycoengineered *Pichia pastoris*. Nat Biotechnol (2006) 24:210–5 10.1038/nbt1178 16429149

[113] Potgieter TI Cukan M Drummond JE Houston-Cummings NR Jiang Y Li F Production of monoclonal antibodies by glycoengineered *Pichia pastoris*. J Biotechnol (2009) 139:318–25 10.1016/j.jbiotec.2008.12.015 19162096

[114] Zhang N Liu L Dumitru CD Cummings NRH Cukan M Jiang Y Glycoengineered *Pichia* produced anti-HER2 is comparable to trastuzumab in preclinical study. MAbs (2011) 3:289–98 10.4161/mabs.3.3.15532 21487242 PMC3149709

[115] Ye J Ly J Watts K Hsu A Walker A McLaughlin K Optimization of a glycoengineered *Pichia pastoris* cultivation process for commercial antibody production. Biotechnol Prog (2011) 27:1744–50 10.1002/btpr.695 22002933

[116] Jiang Y Li F Button M Cukan M Moore R Sharkey N A high-throughput purification of monoclonal antibodies from glycoengineered *Pichia pastoris*. Protein Expr Purif (2010) 74:9–15 10.1016/j.pep.2010.04.016 20447459

[117] Joosten V Lokman C Van Den Hondel CA Punt PJ The production of antibody fragments and antibody fusion proteins by yeasts and filamentous fungi. Microb Cell Fact (2003) 2:1 10.1186/1475-2859-2-1 12605725 PMC149433

[118] Ward M Lin C Victoria DC Fox BP Fox JA Wong DL Characterization of humanized antibodies secreted by *Aspergillus niger*. Appl Environ Microbiol (2004) 70:2567–76 10.1128/AEM.70.5.2567-2576.2004 15128505 PMC404402

[119] Joosten V Gouka RJ van den Hondel CAMJJ Verrips CT Lokman BC Expression and production of llama variable heavy-chain antibody fragments (V(HH)s) by *Aspergillus awamori*. Appl Microbiol Biotechnol (2005) 66:384–92 10.1007/s00253-004-1689-0 15378291

[120] Frenken LG Hessing JG Van den Hondel CA Verrips CT Recent advances in the large-scale production of antibody fragments using lower eukaryotic microorganisms. Res Immunol (1998) 149:589–99 10.1016/S0923-2494(98)80011-4 9835423

[121] Nyyssönen E Penttilä M, Harkki A Saloheimo A Knowles JK Keränen S Efficient production of antibody fragments by the filamentous fungus *Trichoderma reesei*. Biotechnology (N Y) (1993) 11:591–5 10.1038/nbt0593-591 7763606

[122] Joosten V Roelofs MS van den Dries N Goosen T Verrips CT van den Hondel CAMJJ Production of bifunctional proteins by *Aspergillus awamori*: llama variable heavy chain antibody fragment (V(HH)) R9 coupled to *Arthromyces ramosus* peroxidase (ARP). J Biotechnol (2005) 120:347–59 10.1016/j.jbiotec.2005.06.034 16169108

[123] Okazaki F Aoki J Tabuchi S Tanaka T Ogino C Kondo A Efficient heterologous expression and secretion in *Aspergillus oryzae* of a llama variable heavy-chain antibody fragment V(HH) against EGFR. Appl Microbiol Biotechnol (2012) 96:81–8 10.1007/s00253-012-4158-1 22644525

[124] Verdoes JC Punt PJ Burlingame R Bartels J Dijk R van Slump E ORIGINAL RESEARCH: a dedicated vector for efficient library construction and high throughput screening in the hyphal fungus *Chrysosporium lucknowense*. Ind Biotechnol (2007) 3:48–57 10.1089/ind.2007.3.048

[125] Basile G Peticca M Recombinant protein expression in *Leishmania tarentolae*. Mol Biotechnol (2009) 43:273–8 10.1007/s12033-009-9213-5 19779853

[126] Niimi T Recombinant protein production in the eukaryotic protozoan parasite *Leishmania tarentolae*: a review. Methods Mol Biol (2012) 824:307–15 10.1007/978-1-61779-433-9_15 22160905

[127] Klatt S Rohe M Alagesan K Kolarich D Konthur Z Hartl D Production of glycosylated soluble amyloid precursor protein alpha (sAPPalpha) in *Leishmania tarentolae*. J Proteome Res (2013) 12:396–403 10.1021/pr300693f 23214446

[128] Klatt S Konthur Z Secretory signal peptide modification for optimized antibody-fragment expression-secretion in *Leishmania tarentolae*. Microb Cell Fact (2012) 11:97 10.1186/1475-2859-11-97 22830363 PMC3416730

[129] Condreay JP Witherspoon SM Clay WC Kost TA Transient and stable gene expression in mammalian cells transduced with a recombinant baculovirus vector. Proc Natl Acad Sci U S A (1999) 96:127–32 10.1073/pnas.96.1.127 9874783 PMC15104

[130] Hofmann C Sandig V Jennings G Rudolph M Schlag P Strauss M Efficient gene transfer into human hepatocytes by baculovirus vectors. Proc Natl Acad Sci U S A (1995) 92:10099–103 10.1073/pnas.92.22.10099 7479733 PMC40743

[131] Davis TR Wickham TJ McKenna KA Granados RR Shuler ML Wood HA Comparative recombinant protein production of eight insect cell lines. In vitro Cell Dev Biol Anim (1993) 29A:388–90 10.1007/BF02633986 8314732

[132] Kretzschmar T Aoustin L Zingel O Marangi M Vonach B Towbin H High-level expression in insect cells and purification of secreted monomeric single-chain Fv antibodies. J Immunol Methods (1996) 195:93–101 10.1016/0022-1759(96)00093-2 8814324

[133] Liang M Dübel S Li D Queitsch I Li W Bautz EK Baculovirus expression cassette vectors for rapid production of complete human IgG from phage display selected antibody fragments. J Immunol Methods (2001) 247:119–30 10.1016/S0022-1759(00)00322-7 11150543

[134] Hsu TA Takahashi N Tsukamoto Y Kato K Shimada I Masuda K Differential N-glycan patterns of secreted and intracellular IgG produced in *Trichoplusia ni* cells. J Biol Chem (1997) 272:9062–70 10.1074/jbc.272.14.9062 9083032

[135] Jin BR Ryu CJ Kang SK Han MH Hong HJ Characterization of a murine-human chimeric antibody with specificity for the pre-S2 surface antigen of hepatitis B virus expressed in baculovirus-infected insect cells. Virus Res (1995) 38:269–77 10.1016/0168-1702(95)00051-Q 8578864

[136] Zu Putlitz J Kubasek WL Duchêne M Marget M von Specht BU Domdey H Antibody production in baculovirus-infected insect cells. Biotechnology (N Y) (1990) 8:651–4 10.1038/nbt0790-651 1367456

[137] Edelman L Margaritte C Chaabihi H Monchâtre E Blanchard D Cardona A Obtaining a functional recombinant anti-rhesus (D) antibody using the baculovirus-insect cell expression system. Immunology (1997) 91:13–9 10.1046/j.1365-2567.1997.00219.x 9203960 PMC1364029

[138] Ailor E Betenbaugh MJ Modifying secretion and post-translational processing in insect cells. Curr Opin Biotechnol (1999) 10:142–5 10.1016/S0958-1669(99)80024-X 10209136

[139] Aumiller JJ Mabashi-Asazuma H Hillar A Shi X Jarvis DL A new glycoengineered insect cell line with an inducibly mammalianized protein N-glycosylation pathway. Glycobiology (2012) 22:417–28 10.1093/glycob/cwr160 22042767 PMC3267531

[140] Jarvis DL Developing baculovirus-insect cell expression systems for humanized recombinant glycoprotein production. Virology (2003) 310:1–7 10.1016/S0042-6822(03)00120-X 12788624 PMC3641552

[141] Tomiya N Betenbaugh MJ Lee YC Humanization of lepidopteran insect-cell-produced glycoproteins. Acc Chem Res (2003) 36:613–20 10.1021/ar020202v 12924958

[142] Hasemann CA Capra JD High-level production of a functional immunoglobulin heterodimer in a baculovirus expression system. Proc Natl Acad Sci U S A (1990) 87:3942–6 10.1073/pnas.87.10.3942 2111022 PMC54020

[143] Hsu TA Betenbaugh MJ Coexpression of molecular chaperone BiP improves immunoglobulin solubility and IgG secretion from *Trichoplusia ni* insect cells. Biotechnol Prog (1997) 13:96–104 10.1021/bp960088d 9041711

[144] Reavy B Ziegler A Diplexcito J MacIntosh SM Torrance L Mayo M Expression of functional recombinant antibody molecules in insect cell expression systems. Protein Expr Purif (2000) 18:221–8 10.1006/prep.1999.1191 10686153

[145] Seamans TC Gould SL DiStefano DJ Silberklang M Robinson DK Use of lipid emulsions as nutritional supplements in mammalian cell culture. Ann N Y Acad Sci (1994) 745:240–3 10.1111/j.1749-6632.1994.tb44377.x 7832513

[146] Wurm FM Production of recombinant protein therapeutics in cultivated mammalian cells. Nat Biotechnol (2004) 22:1393–8 10.1038/nbt1026 15529164

[147] Jones D Kroos N Anema R van Montfort B Vooys A van der Kraats S High-level expression of recombinant IgG in the human cell line per.c6. Biotechnol Prog (2003) 19:163–8 10.1021/bp025574h 12573020

[148] Butler M Animal cell cultures: recent achievements and perspectives in the production of biopharmaceuticals. Appl Microbiol Biotechnol (2005) 68:283–91 10.1007/s00253-005-1980-8 15834715

[149] Fussenegger M Bailey JE Molecular regulation of cell-cycle progression and apoptosis in mammalian cells: implications for biotechnology. Biotechnol Prog (1998) 14:807–33 10.1021/bp9800891 9841643

[150] Lifely MR Hale C Boyce S Keen MJ Phillips J Glycosylation and biological activity of CAMPATH-1H expressed in different cell lines and grown under different culture conditions. Glycobiology (1995) 5:813–22 10.1093/glycob/5.8.813 8720080

[151] Niklas J Schräder E Sandig V Noll T Heinzle E Quantitative characterization of metabolism and metabolic shifts during growth of the new human cell line AGE1.HN using time resolved metabolic flux analysis. Bioprocess Biosyst Eng (2011) 34:533–45 10.1007/s00449-010-0502-y 21188421 PMC3092918

[152] Shields RL Lai J Keck R O’Connell LY Hong K Meng YG Lack of fucose on human IgG1 N-linked oligosaccharide improves binding to human Fcgamma RIII and antibody-dependent cellular toxicity. J Biol Chem (2002) 277:26733–40 10.1074/jbc.M202069200 11986321

[153] Le Hir H Nott A Moore MJ How introns influence and enhance eukaryotic gene expression. Trends Biochem Sci (2003) 28:215–20 10.1016/S0968-0004(03)00052-5 12713906

[154] Nott A Le Hir H Moore MJ Splicing enhances translation in mammalian cells: an additional function of the exon junction complex. Genes Dev (2004) 18:210–22 10.1101/gad.1163204 14752011 PMC324426

[155] Li J Menzel C Meier D Zhang C Dübel S Jostock T A comparative study of different vector designs for the mammalian expression of recombinant IgG antibodies. J Immunol Methods (2007) 318:113–24 10.1016/j.jim.2006.10.010 17161420

[156] Li J Zhang C Jostock T Dübel S Analysis of IgG heavy chain to light chain ratio with mutant Encephalomyocarditis virus internal ribosome entry site. Protein Eng Des Sel (2007) 20:491–6 10.1093/protein/gzm038 17951613

[157] Omasa T Gene amplification and its application in cell and tissue engineering. J Biosci Bioeng (2002) 94:600–5 10.1016/S1389-1723(02)80201-8 16233356

[158] Peakman TC Worden J Harris RH Cooper H Tite J Page MJ Comparison of expression of a humanized monoclonal antibody in mouse NSO myeloma cells and Chinese hamster ovary cells. Hum Antibodies Hybridomas (1994) 5:65–74 7532024

[159] Spier RE Griffiths JB Berthold W Animal Cell Technology: Products of Today, Prospects for Tomorrow. Oxford: Butterworth-Heinemann Limited (1994).

[160] Zhou W Chen CC Buckland B Aunins J Fed-batch culture of recombinant NS0 myeloma cells with high monoclonal antibody production. Biotechnol Bioeng (1997) 55:783–92 10.1002/(SICI)1097-0290(19970905)55:5 18636588

[161] Jostock T Expression of antibody in mammalian cells. In: Al-Rubeai M editor. Antibody Expression and Production, Cell Engineering. Netherlands: Springer (2011). p. 1–24

[162] Girod P-A Mermod N Use of scaffold/matrix-attachment regions for protein production. In: Makrides SC editor. New Comprehensive Biochemistry. Amsterdam, NL: Elsevier (2003). p. 359–79

[163] Antoniou M Harland L Mustoe T Williams S Holdstock J Yague E Transgenes encompassing dual-promoter CpG islands from the human TBP and HNRPA2B1 loci are resistant to heterochromatin-mediated silencing. Genomics (2003) 82:269–79 10.1016/S0888-7543(03)00107-1 12906852

[164] Kwaks THJ Barnett P Hemrika W Siersma T Sewalt RGAB Satijn DPE Identification of anti-repressor elements that confer high and stable protein production in mammalian cells. Nat Biotechnol (2003) 21:553–8 10.1038/nbt814 12679786

[165] Fernández LA Winkler M Grosschedl R Matrix attachment region-dependent function of the immunoglobulin mu enhancer involves histone acetylation at a distance without changes in enhancer occupancy. Mol Cell Biol (2001) 21:196–208 10.1128/MCB.21.1.196-208.2001 11113195 PMC88794

[166] Zahn-Zabal M Kobr M Girod PA Imhof M Chatellard P de Jesus M Development of stable cell lines for production or regulated expression using matrix attachment regions. J Biotechnol (2001) 87:29–42 10.1016/S0168-1656(00)00423-5 11267697

[167] Gorman CM Howard BH Reeves R Expression of recombinant plasmids in mammalian cells is enhanced by sodium butyrate. Nucleic Acids Res (1983) 11:7631–48 10.1093/nar/11.21.7631 6316266 PMC326508

[168] Classon BJ Brown MH Garnett D Somoza C Barclay AN Willis AC The hinge region of the CD8 alpha chain: structure, antigenicity, and utility in expression of immunoglobulin superfamily domains. Int Immunol (1992) 4:215–25 10.1093/intimm/4.2.215 1377946

[169] Kober L Zehe C Bode J Development of a novel ER stress based selection system for the isolation of highly productive clones. Biotechnol Bioeng (2012) 109:2599–611 10.1002/bit.24527 22510960

[170] Nehlsen K Schucht R da Gama-Norton L Krömer W Baer A Cayli A Recombinant protein expression by targeting pre-selected chromosomal loci. BMC Biotechnol (2009) 9:100 10.1186/1472-6750-9-100 20003421 PMC2804664

[171] Fang J Yi S Simmons A Tu GH Nguyen M Harding TC An antibody delivery system for regulated expression of therapeutic levels of monoclonal antibodies in vivo. Mol Ther (2007) 15:1153–9 17375065 10.1038/sj.mt.6300142

[172] Jostock T Dragic Z Fang J Jooss K Wilms B Knopf H-P Combination of the 2A/furin technology with an animal component free cell line development platform process. Appl Microbiol Biotechnol (2010) 87:1517–24 10.1007/s00253-010-2625-0 20461511

[173] Wilke S Groebe L Maffenbeier V Jäger V Gossen M Josewski J Streamlining homogeneous glycoprotein production for biophysical and structural applications by targeted cell line development. PLoS ONE (2011) 6:e27829 10.1371/journal.pone.0027829 22174749 PMC3235087

[174] Geisse S Fux C Recombinant protein production by transient gene transfer into Mammalian cells. Methods Enzymol (2009) 463:223–38 10.1016/S0076-6879(09)63015-9 19892175

[175] Baldi L Hacker DL Adam M Wurm FM Recombinant protein production by large-scale transient gene expression in mammalian cells: state of the art and future perspectives. Biotechnol Lett (2007) 29:677–84 10.1007/s10529-006-9297-y 17235486

[176] Jostock T Vanhove M Brepoels E Van Gool R Daukandt M Wehnert A Rapid generation of functional human IgG antibodies derived from Fab-on-phage display libraries. J Immunol Methods (2004) 289:65–80 10.1016/j.jim.2004.03.014 15251413

[177] Backliwal G Hildinger M Chenuet S Wulhfard S De Jesus M Wurm FM Rational vector design and multi-pathway modulation of HEK 293E cells yield recombinant antibody titers exceeding 1 g/l by transient transfection under serum-free conditions. Nucleic Acids Res (2008) 36:e96 10.1093/nar/gkn423 18617574 PMC2528171

[178] Geisse S Henke M Large-scale transient transfection of mammalian cells: a newly emerging attractive option for recombinant protein production. J Struct Funct Genomics (2005) 6:165–70 10.1007/s10969-005-2826-4 16211514

[179] Tuvesson O Uhe C Rozkov A Lüllau E Development of a generic transient transfection process at 100 L scale. Cytotechnology (2008) 56:123–36 10.1007/s10616-008-9135-2 19002850 PMC2259262

[180] Meissner P Pick H Kulangara A Chatellard P Friedrich K Wurm FM Transient gene expression: recombinant protein production with suspension-adapted HEK293-EBNA cells. Biotechnol Bioeng (2001) 75:197–203 10.1002/bit.1179 11536142

[181] Boussif O Zanta MA Behr JP Optimized galenics improve in vitro gene transfer with cationic molecules up to 1000-fold. Gene Ther (1996) 3:1074–80 8986433

[182] Thomas M Klibanov AM Enhancing polyethylenimine’s delivery of plasmid DNA into mammalian cells. Proc Natl Acad Sci U S A (2002) 99:14640–5 10.1073/pnas.192581499 12403826 PMC137472

[183] Loignon M Perret S Kelly J Boulais D Cass B Bisson L Stable high volumetric production of glycosylated human recombinant IFNalpha2b in HEK293 cells. BMC Biotechnol (2008) 8:65 10.1186/1472-6750-8-65 18752669 PMC2538527

[184] Zhang J MacKenzie R Durocher Y Production of chimeric heavy-chain antibodies. Methods Mol Biol (2009) 525:323–36 10.1007/978-1-59745-554-1_17 19252853

[185] Daniell H Streatfield SJ Wycoff K Medical molecular farming: production of antibodies, biopharmaceuticals and edible vaccines in plants. Trends Plant Sci (2001) 6:219–26 10.1016/S1360-1385(01)01922-7 11335175 PMC5496653

[186] Hellens RP Edwards EA Leyland NR Bean S Mullineaux PM pGreen: a versatile and flexible binary Ti vector for *Agrobacterium*-mediated plant transformation. Plant Mol Biol (2000) 42:819–32 10.1023/A:1006496308160 10890530

[187] Hoekema A Hirsch PR Hooykaas PJJ Schilperoort RA A binary plant vector strategy based on separation of vir- and T-region of the *Agrobacterium tumefaciens* Ti-plasmid. Nature (1983) 303:179–80 10.1038/303179a0

[188] Amian AA Papenbrock J Jacobsen H-J Hassan F Enhancing transgenic pea (*Pisum sativum* L.) resistance against fungal diseases through stacking of two antifungal genes (chitinase and glucanase). GM Crops (2011) 2:104–9 10.4161/gmcr.2.2.16125 21971070

[189] Horsch RB Fry JE Hofmann NL Eichholz D Rogers SG Fraley RT A simple and general method for transferring genes into plants. Science (1985) 227:1229–31 10.1126/science.227.4691.1229 17757866

[190] McCormick AA Reinl SJ Cameron TI Vojdani F Fronefield M Levy R Individualized human scFv vaccines produced in plants: humoral anti-idiotype responses in vaccinated mice confirm relevance to the tumor Ig. J Immunol Methods (2003) 278:95–104 10.1016/S0022-1759(03)00208-4 12957399

[191] McCormick AA Reddy S Reinl SJ Cameron TI Czerwinkski DK Vojdani F Plant-produced idiotype vaccines for the treatment of non-Hodgkin’s lymphoma: safety and immunogenicity in a phase I clinical study. Proc Natl Acad Sci U S A (2008) 105:10131–6 10.1073/pnas.0803636105 18645180 PMC2481377

[192] Gomord V Chamberlain P Jefferis R Faye L Biopharmaceutical production in plants: problems, solutions and opportunities. Trends Biotechnol (2005) 23:559–65 10.1016/j.tibtech.2005.09.003 16168504

[193] Jin C Altmann F Strasser R Mach L Schähs M Kunert R A plant-derived human monoclonal antibody induces an anti-carbohydrate immune response in rabbits. Glycobiology (2008) 18:235–41 10.1093/glycob/cwm137 18203810

[194] Walsh G Jefferis R Post-translational modifications in the context of therapeutic proteins. Nat Biotechnol (2006) 24:1241–52 10.1038/nbt1252 17033665

[195] Gomord V Denmat LA Fitchette-Lainé AC, Satiat-Jeunemaitre B Hawes C Faye L The C-terminal HDEL sequence is sufficient for retention of secretory proteins in the endoplasmic reticulum (ER) but promotes vacuolar targeting of proteins that escape the ER. Plant J (1997) 11:313–25 10.1046/j.1365-313X.1997.11020313.x 9076996

[196] Schouten A Roosien J de Boer JM Wilmink A Rosso MN Bosch D Improving scFv antibody expression levels in the plant cytosol. FEBS Lett (1997) 415:235–41 10.1016/S0014-5793(97)01129-0 9351003

[197] Schouten A Roosien J van Engelen FA de Jong GA Borst-Vrenssen AW Zilverentant JF The C-terminal KDEL sequence increases the expression level of a single-chain antibody designed to be targeted to both the cytosol and the secretory pathway in transgenic tobacco. Plant Mol Biol (1996) 30:781–93 10.1007/BF00019011 8624409

[198] Schähs M Strasser R Stadlmann J Kunert R Rademacher T Steinkellner H Production of a monoclonal antibody in plants with a humanized N-glycosylation pattern. Plant Biotechnol J (2007) 5:657–63 10.1111/j.1467-7652.2007.00273.x 17678502

[199] Strasser R Stadlmann J Schähs M Stiegler G Quendler H Mach L Generation of glyco-engineered *Nicotiana benthamiana* for the production of monoclonal antibodies with a homogeneous human-like N-glycan structure. Plant Biotechnol J (2008) 6:392–402 10.1111/j.1467-7652.2008.00330.x 18346095

[200] Yin B-J Gao T Zheng N-Y Li Y Tang S-Y Liang L-M Generation of glyco-engineered BY2 cell lines with decreased expression of plant-specific glycoepitopes. Protein Cell (2011) 2:41–7 10.1007/s13238-011-1007-4 21337008 PMC4875288

[201] Vézina L-P Faye L Lerouge P D’Aoust M-A Marquet-Blouin E Burel C Transient co-expression for fast and high-yield production of antibodies with human-like N-glycans in plants. Plant Biotechnol J (2009) 7:442–55 10.1111/j.1467-7652.2009.00414.x 19422604

[202] Jez J Castilho A Grass J Vorauer-Uhl K Sterovsky T Altmann F Expression of functionally active sialylated human erythropoietin in plants. Biotechnol J (2013) 8:371–82 10.1002/biot.201200363 23325672 PMC3601435

[203] Castilho A Neumann L Daskalova S Mason HS Steinkellner H Altmann F Engineering of sialylated mucin-type O-glycosylation in plants. J Biol Chem (2012) 287:36518–26 10.1074/jbc.M112.402685 22948156 PMC3476317

[204] Yang Z Drew DP Jørgensen B Mandel U Bach SS Ulvskov P Engineering mammalian mucin-type O-glycosylation in plants. J Biol Chem (2012) 287:11911–23 10.1074/jbc.M111.312918 22334671 PMC3320939

[205] Mett V Chichester JA Stewart ML Musiychuk K Bi H Reifsnyder CJ A non-glycosylated, plant-produced human monoclonal antibody against anthrax protective antigen protects mice and non-human primates from *B. anthracis* spore challenge. Hum Vaccin (2011) 7:183–90 10.4161/hv.7.0.14586 21270531

[206] Rodríguez M Pérez L Gavilondo JV Garrido G Bequet-Romero M Hernández I Comparative in vitro and experimental in vivo studies of the anti-epidermal growth factor receptor antibody nimotuzumab and its aglycosylated form produced in transgenic tobacco plants. Plant Biotechnol J (2013) 11:53–65 10.1111/pbi.12006 23046448

[207] Marillonnet S Thoeringer C Kandzia R Klimyuk V Gleba Y Systemic *Agrobacterium tumefaciens*-mediated transfection of viral replicons for efficient transient expression in plants. Nat Biotechnol (2005) 23:718–23 10.1038/nbt1094 15883585

[208] Giritch A Marillonnet S Engler C van Eldik G Botterman J Klimyuk V Rapid high-yield expression of full-size IgG antibodies in plants coinfected with noncompeting viral vectors. Proc Natl Acad Sci U S A (2006) 103:14701–6 10.1073/pnas.0606631103 16973752 PMC1566189

[209] Barros GOF Woodard SL Nikolov ZL Phenolics removal from transgenic Lemna minor extracts expressing mAb and impact on mAb production cost. Biotechnol Prog (2011) 27:410–8 10.1002/btpr.543 21485031

[210] Naik AD Menegatti S Reese HR Gurgel PV Carbonell RG Process for purification of monoclonal antibody expressed in transgenic Lemna plant extract using dextran-coated charcoal and hexamer peptide affinity resin. J Chromatogr A (2012) 1260:61–6 10.1016/j.chroma.2012.08.043 22981461

[211] Woodard SL Wilken LR Barros GOF White SG Nikolov ZL Evaluation of monoclonal antibody and phenolic extraction from transgenic Lemna for purification process development. Biotechnol Bioeng (2009) 104:562–71 10.1002/bit.22428 19575415

[212] Torres E Vaquero C Nicholson L Sack M Stöger E Drossard J Rice cell culture as an alternative production system for functional diagnostic and therapeutic antibodies. Transgenic Res (1999) 8:441–9 10.1023/A:1008969031219 10767987

[213] Buck SD Virdi V Meyer TD Wilde KD Piron R Nolf J Production of Camel-like antibodies in plants. In: Saerens D Muyldermans S editors. Single Domain Antibodies, Methods in Molecular Biology. Clifton, NJ: Humana Press (2012). p. 305–24 10.1007/978-1-61779-968-6_19 22886260

[214] De Wilde K De Buck S Vanneste K Depicker A Recombinant antibody production in *Arabidopsis* seeds triggers an unfolded protein response. Plant Physiol (2012) 161:1021–33 10.1104/pp.112.209718 23188806 PMC3561000

[215] Bardor M Loutelier-Bourhis C Paccalet T Cosette P Fitchette A-C Vézina L-P Monoclonal C5-1 antibody produced in transgenic alfalfa plants exhibits a N-glycosylation that is homogenous and suitable for glyco-engineering into human-compatible structures. Plant Biotechnol J (2003) 1:451–62 10.1046/j.1467-7652.2003.00041.x 17134403

[216] He J Lai H Brock C Chen Q A novel system for rapid and cost-effective production of detection and diagnostic reagents of West Nile virus in plants. J Biomed Biotechnol (2012) 2012:106783 10.1155/2012/106783 22187532 PMC3236498

[217] Rademacher T Sack M Arcalis E Stadlmann J Balzer S Altmann F Recombinant antibody 2G12 produced in maize endosperm efficiently neutralizes HIV-1 and contains predominantly single-GlcNAc N-glycans. Plant Biotechnol J (2008) 6:189–201 10.1111/j.1467-7652.2007.00306.x 17979949

[218] Ramessar K Rademacher T Sack M Stadlmann J Platis D Stiegler G Cost-effective production of a vaginal protein microbicide to prevent HIV transmission. Proc Natl Acad Sci U S A (2008) 105:3727–32 10.1073/pnas.0708841104 18316741 PMC2268773

[219] Kirchhoff J Raven N Boes A Roberts JL Russell S Treffenfeldt W Monoclonal tobacco cell lines with enhanced recombinant protein yields can be generated from heterogeneous cell suspension cultures by flow sorting. Plant Biotechnol J (2012) 10:936–44 10.1111/j.1467-7652.2012.00722.x 22758383

[220] Khoudi H Laberge S Ferullo JM Bazin R Darveau A Castonguay Y Production of a diagnostic monoclonal antibody in perennial alfalfa plants. Biotechnol Bioeng (1999) 64:135–43 10.1002/(SICI)1097-0290(19990720)64:2<135::AID-BIT2>3.3.CO;2-H 10397849

[221] Ma JK Hikmat BY Wycoff K Vine ND Chargelegue D Yu L Characterization of a recombinant plant monoclonal secretory antibody and preventive immunotherapy in humans. Nat Med (1998) 4:601–6 10.1038/nm0598-601 9585235

[222] Larrick JW Yu L Chen J Jaiswal S Wycoff K Production of antibodies in transgenic plants. Res Immunol (1998) 149:603–8 10.1016/S0923-2494(98)80013-8 9835425

[223] Wycoff KL Secretory IgA antibodies from plants. Curr Pharm Des (2005) 11:2429–37 10.2174/1381612054367508 16026297

[224] McCormick AA Tobacco derived cancer vaccines for non-Hodgkin’s lymphoma: perspectives and progress. Hum Vaccin (2011) 7:305–12 10.4161/hv.7.3.14163 21346416

[225] Horneff G Becker W Wolf F Kalden JR Burmester GR Human anti-murine immunoglobulin antibodies as disturbing factors in TSH determination. Klin Wochenschr (1991) 69:220–3 10.1007/BF01646945 2033916

[226] Horneff G Winkler T Kalden JR Emmrich F Burmester GR Human anti-mouse antibody response induced by anti-CD4 monoclonal antibody therapy in patients with rheumatoid arthritis. Clin Immunol Immunopathol (1991) 59:89–103 10.1016/0090-1229(91)90084-N 2019013

[227] Castilla J Pintado B Sola I Sánchez-Morgado JM Enjuanes L Engineering passive immunity in transgenic mice secreting virus-neutralizing antibodies in milk. Nat Biotechnol (1998) 16:349–54 10.1038/nbt0498-349 9555725 PMC7097410

[228] Castilla J Sola I Pintado B Sánchez-Morgado JM Enjuanes L Lactogenic immunity in transgenic mice producing recombinant antibodies neutralizing coronavirus. Adv Exp Med Biol (1998) 440:675–86 10.1007/978-1-4615-5331-1_87 9782344

[229] Limonta J Pedraza A Rodríguez A Freyre FM Barral AM Castro FO Production of active anti-CD6 mouse/human chimeric antibodies in the milk of transgenic mice. Immunotechnology (1995) 1:107–13 10.1016/1380-2933(95)00010-0 9373339

[230] Newton DL Pollock D DiTullio P Echelard Y Harvey M Wilburn B Antitransferrin receptor antibody-RNase fusion protein expressed in the mammary gland of transgenic mice. J Immunol Methods (1999) 231:159–67 10.1016/S0022-1759(99)00154-4 10648935

[231] Gavin WG Pollock D Fell P Yelton D Cammuso C Harrington M Expression of the antibody hBR96-2 in the milk of transgenic mice and production of hBR96-2 transgenic goats. Theriogenology (1997) 47:214 10.1016/S0093-691X(97)82341-2

[232] Zhu L van de Lavoir M-C Albanese J Beenhouwer DO Cardarelli PM Cuison S Production of human monoclonal antibody in eggs of chimeric chickens. Nat Biotechnol (2005) 23:1159–69 10.1038/nbt1132 16127450

[233] Brüggemann M Caskey HM Teale C Waldmann H Williams GT Surani MA A repertoire of monoclonal antibodies with human heavy chains from transgenic mice. Proc Natl Acad Sci U S A (1989) 86:6709–13 10.1073/pnas.86.17.6709 2505258 PMC297915

[234] Kuroiwa Y Kasinathan P Choi YJ Naeem R Tomizuka K Sullivan EJ Cloned transchromosomic calves producing human immunoglobulin. Nat Biotechnol (2002) 20:889–94 10.1038/nbt727 12172556

[235] Kuroiwa Y Kasinathan P Matsushita H Sathiyaselan J Sullivan EJ Kakitani M Sequential targeting of the genes encoding immunoglobulin-mu and prion protein in cattle. Nat Genet (2004) 36:775–80 10.1038/ng1373 15184897

[236] Richt JA Kasinathan P Hamir AN Castilla J Sathiyaseelan T Vargas F Production of cattle lacking prion protein. Nat Biotechnol (2007) 25:132–8 10.1038/nbt1271 17195841 PMC2813193

[237] Kuroiwa Y Kasinathan P Sathiyaseelan T Jiao J Matsushita H Sathiyaseelan J Antigen-specific human polyclonal antibodies from hyperimmunized cattle. Nat Biotechnol (2009) 27:173–81 10.1038/nbt.1521 19151699

[238] Grosse-Hovest L Müller S Minoia R Wolf E Zakhartchenko V Wenigerkind H Cloned transgenic farm animals produce a bispecific antibody for T cell-mediated tumor cell killing. Proc Natl Acad Sci U S A (2004) 101:6858–63 10.1073/pnas.0308487101 15105446 PMC406432

[239] Flisikowska T Thorey IS Offner S Ros F Lifke V Zeitler B Efficient immunoglobulin gene disruption and targeted replacement in rabbit using zinc finger nucleases. PLoS ONE (2011) 6:e21045 10.1371/journal.pone.0021045 21695153 PMC3113902

[240] Mendicino M Ramsoondar J Phelps C Vaught T Ball S LeRoith T Generation of antibody- and B cell-deficient pigs by targeted disruption of the J-region gene segment of the heavy chain locus. Transgenic Res (2011) 20:625–41 10.1007/s11248-010-9444-z 20872248 PMC7089184

[241] Ramsoondar J Mendicino M Phelps C Vaught T Ball S Monahan J Targeted disruption of the porcine immunoglobulin kappa light chain locus. Transgenic Res (2011) 20:643–53 10.1007/s11248-010-9445-y 20872247

[242] Houdebine L-M Production of pharmaceutical proteins by transgenic animals. Comp Immunol Microbiol Infect Dis (2009) 32:107–21 10.1016/j.cimid.2007.11.005 18243312 PMC7112688

[243] Green LL Hardy MC Maynard-Currie CE Tsuda H Louie DM Mendez MJ Antigen-specific human monoclonal antibodies from mice engineered with human Ig heavy and light chain YACs. Nat Genet (1994) 7:13–21 10.1038/ng0594-13 8075633

[244] Lonberg N Huszar D Human antibodies from transgenic mice. Int Rev Immunol (1995) 13:65–93 10.3109/08830189509061738 7494109

[245] Pruzina S Williams GT Kaneva G Davies SL Martín-López A Brüggemann M Human monoclonal antibodies to HIV-1 gp140 from mice bearing YAC-based human immunoglobulin transloci. Protein Eng Des Sel (2011) 24:791–9 10.1093/protein/gzr038 21810921

[246] Osborn MJ Ma B Avis S Binnie A Dilley J Yang X High-affinity IgG antibodies develop naturally in Ig-knockout rats carrying germline human IgH/Igκ/Igλ loci bearing the rat CH region. J Immunol (2013) 190:1481–90 10.4049/jimmunol.1203041 23303672 PMC3566577

[247] Chen C Snedecor B Nishihara JC Joly JC McFarland N Andersen DC High-level accumulation of a recombinant antibody fragment in the periplasm of *Escherichia coli* requires a triple-mutant (degP prc spr) host strain. Biotechnol Bioeng (2004) 85:463–74 10.1002/bit.20014 14760686

[248] Monsellier E Bedouelle H Improving the stability of an antibody variable fragment by a combination of knowledge-based approaches: validation and mechanisms. J Mol Biol (2006) 362:580–93 10.1016/j.jmb.2006.07.044 16926023

[249] Rahbarizadeh F Rasaee MJ Forouzandeh-Moghadam M Allameh A-A High expression and purification of the recombinant camelid anti-MUC1 single domain antibodies in *Escherichia coli*. Protein Expr Purif (2005) 44:32–8 10.1016/j.pep.2005.04.008 15922625

[250] Hussack G Arbabi-Ghahroudi M van Faassen H Songer JG Ng KK-S MacKenzie R Neutralization of *Clostridium* difficile toxin A with single-domain antibodies targeting the cell receptor binding domain. J Biol Chem (2011) 286:8961–76 10.1074/jbc.M110.198754 21216961 PMC3058971

[251] Carter P Kelley RF Rodrigues ML Snedecor B Covarrubias M Velligan MD High level *Escherichia coli* expression and production of a bivalent humanized antibody fragment. Biotechnology (N Y) (1992) 10:163–7 1368228 10.1038/nbt0292-163

[252] Cossins AJ Harrison S Popplewell AG Gore MG Recombinant production of a VL single domain antibody in *Escherichia coli* and analysis of its interaction with peptostreptococcal protein L. Protein Expr Purif (2007) 51:253–9 10.1016/j.pep.2006.07.013 16949300

[253] Quintero-Hernández V Juárez-González VR Ortíz-León M Sánchez R Possani LD Becerril B The change of the scFv into the Fab format improves the stability and in vivo toxin neutralization capacity of recombinant antibodies. Mol Immunol (2007) 44:1307–15 10.1016/j.molimm.2006.05.009 16814388

[254] Yang T Yang L Chai W Li R Xie J Niu B A strategy for high-level expression of a single-chain variable fragment against TNFα by subcloning antibody variable regions from the phage display vector pCANTAB 5E into pBV220. Protein Expr Purif (2011) 76:109–14 10.1016/j.pep.2010.10.006 20951213

[255] King DJ Byron OD Mountain A Weir N Harvey A Lawson AD Expression, purification and characterization of B72.3 Fv fragments. Biochem J (1993) 290(Pt 3):723–9 8457200 10.1042/bj2900723 PMC1132340

[256] Ye T Lin Z Lei H High-level expression and characterization of an anti-VEGF165 single-chain variable fragment (scFv) by small ubiquitin-related modifier fusion in *Escherichia coli*. Appl Microbiol Biotechnol (2008) 81:311–7 10.1007/s00253-008-1655-3 18795288 PMC7079844

[257] Nadkarni A Kelley L-LC Momany C Optimization of a mouse recombinant antibody fragment for efficient production from *Escherichia coli*. Protein Expr Purif (2007) 52:219–29 10.1016/j.pep.2006.10.011 17141527

[258] Padiolleau-Lefevre S Alexandrenne C Dkhissi F Clement G Essono S Blache C Expression and detection strategies for an scFv fragment retaining the same high affinity than Fab and whole antibody: implications for therapeutic use in prion diseases. Mol Immunol (2007) 44:1888–96 10.1016/j.molimm.2006.09.035 17140664

[259] Philibert P Stoessel A Wang W Sibler A-P Bec N Larroque C A focused antibody library for selecting scFvs expressed at high levels in the cytoplasm. BMC Biotechnol (2007) 7:81 10.1186/1472-6750-7-81 18034894 PMC2241821

[260] Nesbeth DN Perez-Pardo M-A Ali S Ward J Keshavarz-Moore E Growth and productivity impacts of periplasmic nuclease expression in an *Escherichia coli* Fab’ fragment production strain. Biotechnol Bioeng (2012) 109:517–27 10.1002/bit.23316 21898368

[261] Ueda Y Tsumoto K Watanabe K Kumagai I Synthesis and expression of a DNA encoding the Fv domain of an anti-lysozyme monoclonal antibody, HyHEL10, in *Streptomyces lividans*. Gene (1993) 129:129–34 10.1016/0378-1119(93)90708-B 8335251

[262] Swennen D Paul M-F Vernis L Beckerich J-M Fournier A Gaillardin C Secretion of active anti-Ras single-chain Fv antibody by the yeasts *Yarrowia lipolytica* and *Kluyveromyces lactis*. Microbiology (2002) 148:41–50 11782497 10.1099/00221287-148-1-41

[263] Rahbarizadeh F Rasaee MJ Forouzandeh M Allameh A-A Over expression of anti-MUC1 single-domain antibody fragments in the yeast *Pichia pastoris*. Mol Immunol (2006) 43:426–35 10.1016/j.molimm.2005.03.003 16337485

[264] Ji X Lu W Zhou H Han D Yang L Wu H Covalently dimerized Camelidae antihuman TNFa single-domain antibodies expressed in yeast *Pichia pastoris* show superior neutralizing activity. Appl Microbiol Biotechnol (2013). 10.1007/s00253-012-4639-2 23324801

[265] Ezzine A M’Hirsi el Adab S Bouhaouala-Zahar B Hmila I Baciou L Marzouki MN Efficient expression of the anti-AahI’ scorpion toxin nanobody under a new functional form in a *Pichia pastoris* system. Biotechnol Appl Biochem (2012) 59:15–21 10.1002/bab.67 22332740

[266] Maeng BH Choi J Sa YS Shin JH Kim YH Functional expression of recombinant anti-BNP scFv in methylotrophic yeast *Pichia pastoris* and application as a recognition molecule in electrochemical sensors. World J Microbiol Biotechnol (2012) 28:1027–34 10.1007/s11274-011-0901-5 22805824

[267] Koliasnikov OV Grigorenko VG Egorov AM Lange S Schmid RD Recombinant production of horseradish peroxidase conjugates with Fab antibodies in *Pichia pastoris* for analytical applications. Acta Naturae (2011) 3:85–92 22649698 PMC3347603

[268] Schoonooghe S Kaigorodov V Zawisza M Dumolyn C Haustraete J Grooten J Efficient production of human bivalent and trivalent anti-MUC1 Fab-scFv antibodies in *Pichia pastoris*. BMC Biotechnol (2009) 9:70 10.1186/1472-6750-9-70 19671134 PMC2736937

[269] Gorlani A de Haard H Verrips T Expression of VHHs in *Saccharomyces cerevisiae*. Methods Mol Biol (2012) 911:277–86 10.1007/978-1-61779-968-6_17 22886258

[270] Gorlani A Hulsik DL Adams H Vriend G Hermans P Verrips T Antibody engineering reveals the important role of J segments in the production efficiency of llama single-domain antibodies in *Saccharomyces cerevisiae*. Protein Eng Des Sel (2012) 25:39–46 10.1093/protein/gzr057 22143875

[271] Sommaruga S Lombardi A Salvadè A, Mazzucchelli S Corsi F Galeffi P Highly efficient production of anti-HER2 scFv antibody variant for targeting breast cancer cells. Appl Microbiol Biotechnol (2011) 91:613–21 10.1007/s00253-011-3306-3 21538107

[272] Khatri NK Gocke D Trentmann O Neubauer P Hoffmann F Single-chain antibody fragment production in *Pichia pastoris*: benefits of prolonged pre-induction glycerol feeding. Biotechnol J (2011) 6:452–62 10.1002/biot.201000193 21259439

[273] Jafari R Holm P Piercecchi M Sundström BE Construction of divalent anti-keratin 8 single-chain antibodies (sc(Fv)2), expression in *Pichia pastoris* and their reactivity with multicellular tumor spheroids. J Immunol Methods (2011) 364:65–76 10.1016/j.jim.2010.11.003 21093447

[274] Barnard GC Kull AR Sharkey NS Shaikh SS Rittenhour AM Burnina I High-throughput screening and selection of yeast cell lines expressing monoclonal antibodies. J Ind Microbiol Biotechnol (2010) 37:961–71 10.1007/s10295-010-0746-1 20711797

[275] Wang D Su M Sun Y Huang S Wang J Yan W Expression, purification and characterization of a human single-chain Fv antibody fragment fused with the Fc of an IgG1 targeting a rabies antigen in *Pichia pastoris*. Protein Expr Purif (2012) 86:75–81 10.1016/j.pep.2012.08.015 22982755

[276] Chen M-T Lin S Shandil I Andrews D Stadheim TA Choi B-K Generation of diploid *Pichia pastoris* strains by mating and their application for recombinant protein production. Microb Cell Fact (2012) 11:91 10.1186/1475-2859-11-91 22748191 PMC3503796

[277] Sotiriadis A Keshavarz T Keshavarz-Moore E Factors affecting the production of a single-chain antibody fragment by *Aspergillus awamori* in a stirred tank reactor. Biotechnol Prog (2001) 17:618–23 10.1021/bp010026+ 11485420

[278] Gómez-Sebastián S Nuñez MC Garaicoechea L Alvarado C Mozgovoj M Lasa R Rotavirus A-specific single-domain antibodies produced in baculovirus-infected insect larvae are protective in vivo. BMC Biotechnol (2012) 12:59 10.1186/1472-6750-12-59 22953695 PMC3444942

[279] Kurasawa JH Shestopal SA Jha NK Ovanesov MV Lee TK Sarafanov AG Insect cell-based expression and characterization of a single-chain variable antibody fragment directed against blood coagulation factor VIII. Protein Expr Purif (2013) 88:201–6 10.1016/j.pep.2012.12.008 23306063

[280] Backovic M Johansson DX Klupp BG Mettenleiter TC Persson MAA Rey FA Efficient method for production of high yields of Fab fragments in *Drosophila* S2 cells. Protein Eng Des Sel (2010) 23:169–74 10.1093/protein/gzp088 20100703

[281] Johansson DX Drakenberg K Hopmann KH Schmidt A Yari F Hinkula J Efficient expression of recombinant human monoclonal antibodies in *Drosophila* S2 cells. J Immunol Methods (2007) 318:37–46 10.1016/j.jim.2006.08.017 17137589

[282] Gilmartin AA Lamp B Rümenapf T Persson MAA Rey FA Krey T High-level secretion of recombinant monomeric murine and human single-chain Fv antibodies from *Drosophila* S2 cells. Protein Eng Des Sel (2012) 25:59–66 10.1093/protein/gzr058 22160929 PMC3258843

[283] Palmberger D Rendic D Tauber P Krammer F Wilson IBH Grabherr R Insect cells for antibody production: evaluation of an efficient alternative. J Biotechnol (2011) 153:160–6 10.1016/j.jbiotec.2011.02.009 21477625

[284] Nettleship JE Ren J Rahman N Berrow NS Hatherley D Neil Barclay A A pipeline for the production of antibody fragments for structural studies using transient expression in HEK 293T cells. Protein Expr Purif (2008) 62:83–9 10.1016/j.pep.2008.06.017 18662785

[285] Codamo J Munro TP Hughes BS Song M Gray PP Enhanced CHO cell-based transient gene expression with the epi-CHO expression system. Mol Biotechnol (2011) 48:109–15 10.1007/s12033-010-9351-9 21104043

[286] Van Berkel PHC Gerritsen J van Voskuilen E Perdok G Vink T van de Winkel JGJ Rapid production of recombinant human IgG with improved ADCC effector function in a transient expression system. Biotechnol Bioeng (2010) 105:350–7 10.1002/bit.22535 19739094

[287] Wulhfard S Baldi L Hacker DL Wurm F Valproic acid enhances recombinant mRNA and protein levels in transiently transfected Chinese hamster ovary cells. J Biotechnol (2010) 148:128–32 10.1016/j.jbiotec.2010.05.003 20510314

[288] Backliwal G Hildinger M Kuettel I Delegrange F Hacker DL Wurm FM Valproic acid: a viable alternative to sodium butyrate for enhancing protein expression in mammalian cell cultures. Biotechnol Bioeng (2008) 101:182–9 10.1002/bit.21882 18454496

[289] Wulhfard S Tissot S Bouchet S Cevey J de Jesus M Hacker DL Mild hypothermia improves transient gene expression yields several fold in Chinese hamster ovary cells. Biotechnol Prog (2008) 24:458–65 10.1021/bp070286c 18220408

[290] Mader A Prewein B Zboray K Casanova E Kunert R Exploration of BAC versus plasmid expression vectors in recombinant CHO cells. Appl Microbiol Biotechnol (2012) 97(9):4049–54 10.1007/s00253-012-4498-x 23081777

[291] Kober L Zehe C Bode J Optimized signal peptides for the development of high expressing CHO cell lines. Biotechnol Bioeng (2013) 110:1164–73 10.1002/bit.24776 23124363

[292] Spens E Häggström L Defined protein and animal component-free NS0 fed-batch culture. Biotechnol Bioeng (2007) 98:1183–94 10.1002/bit.21509 17516495

[293] Burky JE Wesson MC Young A Farnsworth S Dionne B Zhu Y Protein-free fed-batch culture of non-GS NS0 cell lines for production of recombinant antibodies. Biotechnol Bioeng (2007) 96:281–93 10.1002/bit.21060 16933323

[294] Tchoudakova A Hensel F Murillo A Eng B Foley M Smith L High level expression of functional human IgMs in human PER.C6 cells. MAbs (2009) 1:163–71 10.4161/mabs.1.2.7945 20061826 PMC2725415

[295] Agrawal V Slivac I Perret S Bisson L St-Laurent G Murad Y Stable expression of chimeric heavy chain antibodies in CHO cells. Methods Mol Biol (2012) 911:287–303 10.1007/978-1-61779-968-6_18 22886259

[296] Huang L Li M-X Lei Y Wang Y-T Xie K Yang Y-L Expression, purification and activity determination of humanized anti-HER2 monoclonal antibody in CHO. Chin Pharm J (2012) 47:884–8

[297] Tran M Van C Barrera DJ Pettersson PL Peinado CD Bui J Production of unique immunotoxin cancer therapeutics in algal chloroplasts. Proc Natl Acad Sci U S A (2013) 110:E15–22 10.1073/pnas.1214638110 23236148 PMC3538218

[298] Almquist KC McLean MD Niu Y Byrne G Olea-Popelka FC Murrant C Expression of an anti-botulinum toxin A neutralizing single-chain Fv recombinant antibody in transgenic tobacco. Vaccine (2006) 24:2079–86 10.1016/j.vaccine.2005.11.014 16337316

[299] Sainsbury F Sack M Stadlmann J Quendler H Fischer R Lomonossoff GP Rapid transient production in plants by replicating and non-replicating vectors yields high quality functional anti-HIV antibody. PLoS ONE (2010) 5:e13976 10.1371/journal.pone.0013976 21103044 PMC2980466

[300] Cui L Peng H Zhang R Chen Y Zhao L Tang K Recombinant hHscFv–RC-RNase protein derived from transgenic tobacco acts as a bifunctional molecular complex against hepatocellular carcinoma. Biotechnol Appl Biochem (2012) 59:323–9 10.1002/bab.1039 23586908

[301] Huang Z Phoolcharoen W Lai H Piensook K Cardineau G Zeitlin L High-level rapid production of full-size monoclonal antibodies in plants by a single-vector DNA replicon system. Biotechnol Bioeng (2010) 106:9–17 10.1002/bit.22652 20047189 PMC2905544

[302] Zhang R Cui D Wang H Li C Yao X Zhao Y Functional recombinant human anti-HBV antibody expressed in milk of transgenic mice. Transgenic Res (2012) 21:1085–91 10.1007/s11248-012-9589-z 22286336

[303] Wei J Yang X Zheng M Wang M Dai Y Chen Z The recombinant chimeric antibody chHAb18 against hepatocellular carcinoma can be produced in milk of transgenic mice. Transgenic Res (2011) 20:321–30 10.1007/s11248-010-9408-3 20549347

[304] Zhang R Rao M Li C Cao J Meng Q Zheng M Functional recombinant human anti-HAV antibody expressed in milk of transgenic mice. Transgenic Res (2009) 18:445–53 10.1007/s11248-008-9241-0 19130282 PMC7089081

[305] Yuskevich V Khodarovich Y Kagarliskiy G Stremovskiy O Maksimenko O Lukash S Expression of humanized anti-Her2/neu single-chain IgG1-like antibody in mammary glands of transgenic mice. Biochimie (2011) 93:628–30 10.1016/j.biochi.2010.12.001 21146579

[306] Kamihira M Kawabe Y Shindo T Ono K Esaka K Yamashita T Production of chimeric monoclonal antibodies by genetically manipulated chickens. J Biotechnol (2009) 141:18–25 10.1016/j.jbiotec.2009.02.022 19428726

